# Characterization of Amphetamine, Methylphenidate, Nicotine, and Atomoxetine on Measures of Attention, Impulsive Action, and Motivation in the Rat: Implications for Translational Research

**DOI:** 10.3389/fphar.2020.00427

**Published:** 2020-04-24

**Authors:** Guy A. Higgins, Leo B. Silenieks, Cam MacMillan, Sandy Thevarkunnel, Anna I. Parachikova, Cedric Mombereau, Hanna Lindgren, Jesper F. Bastlund

**Affiliations:** ^1^ Intervivo Solutions, Toronto, ON, Canada; ^2^ Department of Pharmacology & Toxicology, University of Toronto, Toronto, ON, Canada; ^3^ Discovery Research, H. Lundbeck A/S, Copenhagen, Denmark

**Keywords:** attention, impulsivity, plasma exposure, translation, attentional deficit hyperactivity disorder, rat, five-choice serial reaction time task, Go/NoGo

## Abstract

Amphetamine (AMP), methylphenidate (MPH), and atomoxetine (ATX) are approved treatments for ADHD, and together with nicotine (NIC), represent pharmacological agents widely studied on cognitive domains including attention and impulsive action in humans. These agents thus represent opportunities for clinical observation to be reinvestigated in the preclinical setting, i.e., reverse translation. The present study investigated each drug in male, Long Evans rats trained to perform either (1) the five-choice serial reaction time task (5-CSRTT), (2) Go/NoGo task, or (3) a progressive ratio (PR) task, for the purpose of studying each drug on attention, impulsive action and motivation. Specific challenges were adopted in the 5-CSRTT designed to tax attention and impulsivity, i.e., high frequency of stimulus presentation (sITI), variable reduction in stimulus duration (sSD), and extended delay to stimulus presentation (10-s ITI). Initially, performance of a large (> 80) cohort of rats in each task variant was conducted to examine performance stability over repeated challenge sessions, and to identify subgroups of “high” and “low” attentive rats (sITI and sSD schedules), and “high” and “low” impulsives (10-s ITI). Using an adaptive sequential study design, the effects of AMP, MPH, ATX, and NIC were examined and contrasting profiles noted across the tests. Both AMP (0.03–0.3 mg/kg) and MPH (1–6 mg/kg) improved attentional performance in the sITI but not sSD or 10-s ITI condition, NIC (0.05–0.2 mg/kg) improved accuracy across all conditions. ATX (0.1–1 mg/kg) detrimentally affected performance in the sITI and sSD condition, notably in “high” performers. In tests of impulsive action, ATX reduced premature responses notably in the 10-s ITI condition, and also reduced false alarms in Go/NoGo. Both AMP and NIC increased premature responses in all task variants, although AMP reduced false alarms highlighting differences between these two measures of impulsive action. The effect of MPH was mixed and appeared baseline dependent. ATX reduced break point for food reinforcement suggesting a detrimental effect on motivation for primary reward. Taken together these studies highlight differences between AMP, MPH, and ATX which may translate to their clinical profiles. NIC had the most reliable effect on attentional accuracy, whereas ATX was reliably effective against all tests of impulsive action.

## Introduction

Limitations to the translation of preclinical findings to the clinic has been a longstanding issue which has lead some to question the value of animal models particularly in therapeutic areas such as psychiatry where etiology is generally considered to be poorly understood (Kola and Landis, 2004; Littman and Williams, 2005; Enna and Williams, 2009; van der Worp et al., 2010). One reaction to this view has been to place emphasis on translational research where animal tests are designed to align closely to those conducted in humans, and conversely to design early phase human tests taking features from existing animal tests. A closer correspondence between preclinical and clinical measures should enhance predictability and promote translation back and forth between animal and human studies (Pangalos et al., 2007; Conn and Roth, 2008; Day et al., 2008; Miczek and de Wit, 2008; Markou et al., 2009; Goetghebeur and Swartz, 2016; McArthur, 2017).

A second outcome has been to question the rigor and standards by which animal studies themselves have been conducted with calls for better study design and conduct, and ensuring studies are appropriately powered (Kilkenny et al., 2009; Kilkenny et al., 2010; van der Worp et al., 2010; Button et al., 2013; Bespalov et al., 2016; Bespalov and Steckler, 2018). A further development has been a recognition that psychiatric disorders typically consist of specific symptoms, or endophenotypes, that can extend across multiple diagnostic categories and represent discrete measurable entities (Castellanos and Tannock, 2002; Hyman and Fenton, 2003; Hasler et al., 2004; Robbins et al., 2012; Kozak and Cuthbert, 2016). These endophenotypes include constructs such as attention, impulsivity, working memory, motivation which can be objectively measured in animals using appropriate tests and experimental conditions. Thus complex clinical disorders can be fractionated into discrete symptom clusters which may be more amenable to translational research and treatment (Pangalos et al., 2007; Day et al., 2008; Miczek and de Wit, 2008; Markou et al., 2009; McArthur, 2017).

ADHD is a psychiatric condition which serves as a useful avenue for translational research (Robbins, 2017; Phillips et al., 2018). Firstly, it can be argued that ADHD is the only psychiatric condition for which there are effective drugs to treat the central cognitive symptoms. Methylphenidate (MPH; e.g. Ritalin^®^), amphetamine (AMP; e.g. Adderall^®^), and atomoxetine (ATX; e.g. Strattera^®^) have each been approved as treatments for both juvenile and adult ADHD on the basis of significant efficacy against at least some of the core neurocognitive symptoms (Faraone and Glatt, 2010; Castells et al., 2011a; Castells et al., 2011b; Asherson et al., 2014; Bolea-Alamañac et al., 2014; Epstein et al., 2014; Storebø et al., 2015). Secondly, the neurocognitive symptoms of ADHD such as inattention and impulsivity represent endophenotypes that can be reliably measured both in animals and humans with analogous cross-species tests. For these reasons ADHD presents opportunities both for forward translation from preclinical to clinical trial, and reverse translation for clinical observation to be reinvestigated in the preclinical setting (Winstanley et al., 2006; Chamberlain et al., 2011; Robbins, 2017). Nicotine (NIC) also has well described effects on attention and impulsivity in both the preclinical and clinical setting, and provides a further useful benchmark for translational research (Mirza and Stolerman, 1999; Spinelli et al., 2006; Heishman et al., 2010; Myers et al., 2013). Therapeutics based on targeting nicotinic cholinergic receptors have also been proposed as potential treatments for multiple neuropsychiatric disorders, including ADHD (Levin et al., 1996; Levin et al., 1998; Bain et al., 2013; Potter et al., 2014), although as yet none have reached approval status.

Multiple tests have been developed to measure attention and impulsivity in the rodent and primate which have human counterparts. Reflecting the multifactorial nature of both constructs, multiple tests or adaptive test configurations are necessary to study each domain. The five-choice serial reaction time task (5-CSRTT) developed as a preclinical equivalent to the human continuous performance task (CPT) (Robbins, 2002), has become widely used to measure attention and impulsive action in rodent and primate species (Weed et al., 1999; Spinelli et al., 2004). A strength of the 5-CSRTT is the capability to modify task conditions to differentially challenge attention and response control (Robbins, 2002; Bari et al., 2008; Higgins and Silenieks, 2017). The five-choice CPT (5-CPT) is a closer rodent analog of the human CPT, for unlike the 5-CSRTT, the test includes nontarget stimuli to which the animal must withhold responding (Lustig et al., 2014; Young et al., 2009; Barnes et al., 2012). The Go-NoGo task has both target (or “go”) and nontarget (or “nogo”) trials, and is used both in the preclinical and clinical setting to measure response inhibition (defined as inhibition of a preplanned response; Eagle et al., 2008). Because impairments to response inhibition are considered an important marker of ADHD (Castellanos and Tannock, 2002; Aron and Russell, 2005; Lijffijt et al., 2005), we included the Go-NoGo task in the current test battery.

The current report describes a series of studies designed to characterize the effect of AMP, MPH, NIC, and ATX on performance in the rat 5-CSRTT and Go-NoGo task for the purpose of evaluating these drugs on attention and impulsivity and translating these findings to clinical experience. Three test configurations of the 5-CSRTT were utilized, each designed to differentially tax aspects of attention and impulsive action. In the initial part of this report, the performance of a cohort of rats across these different challenge conditions is described. Due to the large number of animals tested, an assessment of extreme phenotypes relevant to (in)attention and impulsive action could also be determined raising the potential to study drug effects in these subgroups (Puumala et al., 1996; Blondeau and Dellu-Hagedorn, 2007; Hayward et al., 2016). We decided to select subgroups on the basis of tertiles, thus providing some separation between the extreme groups, while recognizing the 3Rs principals of animal research (Hayward et al., 2016). Furthermore, the reliability of key performance measures was determined over repeated challenge sessions. Motivation for the primary reward of these tasks is a critical determinant in overall performance and for this reason the effect of each drug on responding for food under a progressive schedule of reinforcement (Hodos, 1961; Der-Avakian et al., 2016) was also assessed.

## Methods

### Animals and Housing

Adult, male Long Evans rats were used in all studies (Charles River, St. Constant, Quebec, Canada). Animals weighed approximately 250 g upon arrival at the test facility and were singly housed in polycarbonate cages with sawdust bedding and plastic drainpipe for enrichment. Water was freely available; food availability was restricted to that earned during the test session and a preweighed amount of food 16–20 g, adjusted depending on whether animals had been tested that day. Animals were housed in a climatically controlled holding room (room temperature: 22°C ± 2°C) under a 12-h light-dark cycle (lights on: 06:00–18:00 h). All testing was conducted during the light phase of the animal’s light/dark cycle.

All studies were conducted at the InterVivo Solutions test facility. Since most rats were used in multiple studies the age at time of testing ranged from 4 months to 14 months. The studies were approved by the appropriate Institute Animal Care and Use committee and conducted in accordance with guidelines established by the Canadian Council of Animal Care (CCAC). Animal health of all rats was routinely checked by trained veterinary staff and only animals considered to be of good health entered each study.

### Drugs and Injections

d-Amphetamine sulfate (Toronto Research Chemicals; AMP), methylphenidate hydrochloride (Toronto Research Chemicals; MPH), and atomoxetine hydrochloride (H. Lundbeck A/S, Valby, Copenhagen; ATX) were administered intraperitoneally in a dose volume of 1 ml/kg. Nicotine ditartrate dihydrate (Toronto Research Chemicals; NIC) was administered subcutaneously in a dose volume of 1 ml/kg. All drugs were dissolved in a vehicle of 0.9% saline. Final doses of each drug are expressed in terms of the free base. Pretreatment times were 30 min (MPH, ATX), 10 min (AMP, NIC). All rats received a preexposure to a test article prior to testing, for the purpose of minimizing any novel drug interoceptive state influencing behavior.

### Five-Choice Serial Reaction Time Task

Five-choice operant chambers (Med Associates Inc., St. Albans, VT) housed in sound-insulated and ventilated enclosures were used. Each chamber consisted of an aluminum enclosure (25 cm × 30 cm), containing on one wall a reward magazine attached to a food pellet dispenser, and house light, and on the opposite wall an array of 5 square inches (2.5 cm × 2.5 cm × 2.5 cm) arranged on a curved panel and raised 2.5 cm from the grid floor. An LED was positioned at the rear of each niche. Each niche, and the reward magazine, also contained a photocell to detect head entry. Test chambers were controlled by Med PC software (Med Associates Inc., St. Albans, VT).

Training commenced with a couple of sessions in which the food hopper and each light niche were filled with approximately five pellets (45 mg food pellet, Bioserv, USA) each. The 5-CSRTT began with the illumination of the house light and delivery of a food pellet. A nose-poke into the magazine tray initiated the first trial which consisted of an intertrial interval (ITI, 5 s) followed by the random illumination of one of the five lights for a fixed interval (stimulus duration, SD). If a nose-poke was registered in the illuminated niche before the end of either the SD, or a fixed interval after this period (limited hold, LH) a further pellet was dispensed and a Correct Trial registered. An incorrect nose poke (Incorrect Trial) or failure to respond within the allotted time (Missed Trial) resulted in a Time Out (TO) period in which the houselight was extinguished for 5 s. Responding into one of the five niches during the ITI (premature response) resulted in a further TO. Data for perseverative responses (which were unpunished) was not routinely collected across these experiments and so this data is not included. Finally, if a rat responded into a niche during a TO, the TO was restarted.

Each session ran for either 100 trials or 60 min, whichever was shorter. Initially, stimulus parameters were such that SD was set at 60 s, and ITI, TO, and LH were 5 s. For all subjects the SD was progressively reduced until a final duration of 0.75 s was achieved. All other parameters remained at their initial levels throughout training and test, except ITI (see below). Training continued under the target stimulus parameters until subjects had achieved consistent performance above a threshold of 80% correct ([correct/(correct + incorrect)]*100) and <20% omissions for at least a 2-week period. At this point, drug testing began according to a repeated measures design with animals tested twice weekly (either Tuesday/Friday or Wednesday/Saturday) and run under baseline conditions over intervening days.

On test days only, the rats were subjected to three different types of test conditions which were designed to provide distinct challenges to the rats (see Introduction). Specifically the challenge session types were: (A) multiple short ITI (sITI), i.e., 40 presentations each of ITI 2, 3.5, 5 s; 0.3-s stimulus duration, 5-s limited hold, 120 trials total, (B) multiple short stimulus duration (sSD), i.e., 30 presentations each of SD 0.03, 0.1, 0.3, 1 s; 5-s ITI; 5-s limited hold; 120 trials total, (C) long 10-s ITI (10sITI) i.e. 0.3-s stimulus duration, 10-s ITI, 5-s limited hold, 100 trials total. For each of the challenge session types, three levels of analyses were conducted. (A) A meta-analysis of all animals tested under that specific schedule. This included data pooled from different cohorts of rats ran on separate occasions but all rats being tested under identical experimental conditions. (B) Based on the meta-analysis, a key variable was selected and animals were ranked based on performance under that variable. A “low” and “high” performance group were identified based on the lower and upper tertiles. (C) Performance over repeated challenge sessions to establish stability of performance. Having established conditions under which baseline performance remained stable over repeated challenge sessions, the effect of AMP (0.03–0.3 mg/kg), MPH (1–6 mg/kg), NIC (0.05–0.2 mg/kg) and ATX (0.1–1 mg/kg) was investigated on performance under each challenge.

### Go-NoGo Task

Operant test chambers (Med Associates Inc., St. Albans, VT) were housed in sound-insulated and ventilated enclosures. Each chamber consisted of an aluminum enclosure (25 × 30 cm), containing on one wall a food hopper and house light, with a response lever and a stimulus light positioned each side of the hopper. Chambers were controlled by Med PC software using programs written in-house (Med Associates Inc., St. Albans, VT).

Male Long-Evans rats were trained based on the methods described by Kolokotroni et al. (2011). Briefly, the rats were initially trained to lever press for food reward (45-mg food pellet). Following acquisition of the lever press response, rats were trained to a symmetrically reinforced Go/NoGo (lever press/no lever press) conditional visual discrimination task in response to a stimulus light cue (fast 0.1 s/5 Hz or slow 0.75 s/0.5 Hz) to receive food reward, i.e., to Go or NoGo. The visual stimuli being modified slightly from Kolokotroni et al. (2011) to increase their discriminability. Visual stimuli were paired equally between trial type, i.e. fast/Go and slow/NoGo, and slow/Go and fast/NoGo. A test session consisted of 40 Go and 40 NoGo trials presented in a random sequence, each lasting 10 s (approximate session duration 20 min). Rats were trained over a period of 2–3 months, with some rats receiving occasional correction trial sessions to assist task acquisition. On these occasions, the rats received exclusively a NoGo session. Prior to drug testing, all rats had been on the full task schedule, i.e., Go/NoGo, for at least 2 weeks.

The primary measure was the animals’ efficiency in terms of correct responses/total responses made during the Go and NoGo periods. False alarms reflect lever press responses made during a NoGo trial, and failure to correctly respond during a Go trial was recorded as an error. Response latencies were also recorded. Having established conditions under which baseline performance remained stable over repeated challenge sessions, the effect of AMP (0.1–0.6 mg/kg), MPH (1–6 mg/kg), NIC (0.05–0.4 mg/kg), and ATX (0.1–1 mg/kg) was investigated on performance.

### PR Schedule of Food Reinforcement

Using the same test chambers as for the Go-NoGo task, following acquisition of the lever press behavior, 12 rats were trained to respond for food on a single lever under a PR schedule in which the number of responses required to obtain a food pellet increased for successive reinforcers. Responses for successive reinforcers increased according to the progression 2, 4, 6, 9, 12, 15, 20, 25, 32, 40, 50, 62, 77, 95, 118, 145, 178, etc. This sequence was derived from the equation: ratio = [5 × e(0.2 × reinforcer #) – 5]. A rat was assumed to have reached the break point if it failed to receive a reward for 20 min. Drug testing began once rats performed at asymptote, i.e., individual break point did not vary by >15% over three consecutive sessions, which required 2–3 weeks of training. The number or reinforcers earned, i.e. break-point, and the total number of responses made was recorded. Once baseline performance remained stable over repeated challenge sessions, the effect of AMP (0.03–0.6 mg/kg), MPH (1–6 mg/kg), NIC (0.05–0.4 mg/kg), and ATX (0.1–1 mg/kg) was investigated on performance.

### Assessment of Locomotor Activity

Separate groups of rats were utilized for each drug (N=15 per drug). The test subjects were first given sham vehicle injections and two habituation sessions to the test apparatus (17′W × 17′L × 12′H; Med Associates Inc., St. Albans, VT) before testing the effect of the drug on motor behavior over a 90 min session. A repeated measures design was used with a washout period of 2–3 days between each treatment cycle. Total distance traveled, ambulatory, and rearing counts for the total session was recorded as the primary measure. Distance traveled was also collected into 10-min time bins. The effect of AMP (0.03–2 mg/kg), MPH (0.3–6 mg/kg), NIC (0.05–0.4 mg/kg), and ATX (0.1–2 mg/kg) was investigated.

### Assessment of Drug Plasma Levels

Study animals were dosed with either AMP (0.03–0.6 mg/kg), MPH (1–10 mg/kg), NIC (0.05–0.4 mg/kg), or ATX (0.1–2 mg/kg) and blood collected by saphenous draw at timepoints based on pretreatment times that corresponded to behavioral testing in the five-choice and Go/NoGo tasks, i.e. 0.5, 1, and 2 h. Animals were on the same food restriction schedule as used for the behavioral tests. Animals were not behaviorally tested on days of plasma collection.

Bioanalysis was conducted using liquid chromatography-tandem mass spectrometry (LC-MS/MS) systems located either at Lundbeck, Valby DK (AMP, MPH, ATX), or InterVivo Solutions, Toronto, CAN (NIC).

### Analysis of Data

Data from the 5-CSRTT task was analyzed by one (treatment), or two way (treatment × trial type) repeated measures ANOVA (Statistica Version 11, Statsoft Inc. [2012]). In the event of a significant main effect, *post hoc* comparisons were carried out with Dunnett’s test for comparison of drug treatment to vehicle control. A subgroup analysis was also conducted on data collected from the five-choice experiments. Test subjects were divided into high and low performers based either (1) on % hit performance measured under the most challenging test condition (i.e., 2-s ITI = sITI challenge; or 0.03 s SD = sSD challenge), or (2) number of premature responses measured under the long 10-s ITI schedule. Each low and high group consisted of the extreme tertile rats; the middle tertile group was excluded from this analysis. To examine the effect of tertile groups on performance measures, a two way ANOVA (tertile group × trial type) or three-way (treatment × tertile group × trial type) was conducted. To account for any treatment and/or task differences in trial number, premature responses were calculated both as total number and % of trial number. In all cases the accepted level of significance was P < 0.05. Effect sizes for group mean differences were also calculated using partial eta squared (Statistica Version 11, Statsoft Inc. [2012]).

For the Go/NoGo task, the primary measures were % correct under “Go” and “NoGo” condition, the Total % correct (i.e. combined accuracy under “Go” and “NoGo’) and false alarms, i.e. incorrect responses under “NoGo” condition. Response latency measures were also collected. Data were analyzed by one way (treatment) or two way (treatment × trial type) repeated measures ANOVA. PR measures of number of active lever presses, break point and total session duration were collected and analyzed by one way (treatment) ANOVA. Effect sizes for group mean differences were also calculated using partial eta squared (Statistica Version 11, Statsoft Inc. [2012]).

## Results

### Five-Choice Serial Reaction Time Task

#### Standard Test Conditions

Prior to presentation of challenge sessions and drug testing, all rats (N=137) were trained to asymptotic levels of performance under the standard training conditions of SD=0.75 s, ITI=5 s, limited hold=5 s, 100 trials. Under these conditions correct accuracy was approximately 90% (90.5 ± 0.7%), the latency to make a correct response approximately 0.6 s (0.62 ± 0.01 s), and the level of premature responses were approximately 10% of total trial number (N=9.2 ± 0.8, %=9.4 ± 0.8), which typically was 100, i.e. rats completed all trials. Omissions were approximately 10% (10.9 ± 0.9).

#### sITI Test Challenge

##### Characterization of Performance Under the sITI Task

**Figure 1:**
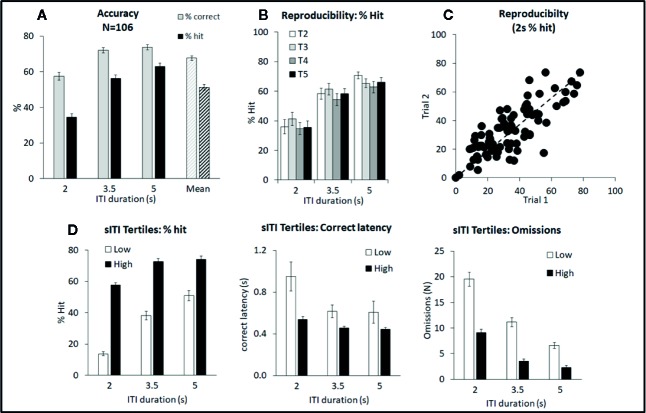
Summary of performance of adult male Long Evans rats in the short intertrial interval (sITI) five-choice schedule. Summary of performance of adult male Long Evans rats in the sITI (2–5 s ITI, 0.3 s SD) 5-CSRTT schedule. **(A)** Meta-analysis of 106 rats tested under sITI schedule—comparison of performance for accuracy (% correct and % hit) at each ITI, and overall % accuracy collapsed across ITI (Mean). **(B)** Reproducibility of performance (% hit) following repeated exposures to test challenge (T2–T5). **(C)** Rats show a wide variation in terms of % hit under the 2-s ITI. These differences are reasonably consistent across repeated testing. **(D)** Ranking rats according to % hit score under the 2-s ITI. Low tertile group (N=35; “Low”) and high tertile group (N=35; “High”). In addition to higher accuracy, the “High” performer group have faster response latencies and make fewer omission errors compared to the “Low” performer subgroup.

The sITI test condition was designed to challenge the test subjects by presenting stimuli at a higher frequency, and with temporal unpredictability, compared to standard conditions, i.e. standard: ITI 5 s; sITI: ITI 2, 3.5, 5 s. Also stimulus duration was shorter placing more challenge on detectability, i.e. standard: SD=0.75 s; sITI: SD=0.3 s. A total of 106 rats were run in this task and a meta-analysis of data from all rats is presented in **Figures 1A–C** and **Table 1**. Performance of rats in this test variant demonstrated the challenge of shortening the ITI with accuracy, response speed, and omissions showing a reliable decline as the ITI decreased from 5 s to 3.5 s to 2 s. Overall, there was a significant main effect of ITI on % correct (F2,210 = 99.6; P < 0.001; η_p_^2^ = 0.29), % hit (F2,210 = 197.9; P < 0.001; η_p_^2^ = 0.65), correct latency (F2,210 = 14.3; P < 0.001; η_p_^2^ = 0.12), omissions (F2,210 = 236.2; P < 0.001; η_p_^2^ = 0.69), and premature responses (F2,210 = 95.2; P < 0.001; η_p_^2^ = 0.48). Because omissions were directly related to task difficulty, i.e., most prevalent at the 2-s ITI, they were included in the accuracy measure—consequently % hit was used as the principal accuracy measure in the sITI task. Although premature responses were significantly affected by ITI, when corrected for trial number they were lower (~5%) than the overall levels recorded during standard test conditions (9.4% ± 0.8%) and so in the context of these experiments considered relatively unimportant.

**Table 1:** Summary of baseline performance and effect of amphetamine (AMP), methylphenidate (MPH), nicotine (NIC), and atomoxetine (ATX) on performance of rats in the short intertrial interval (sITI) schedule.

	Dose (mg/kg)	N	2s ITI		3.5s ITI		5s ITI		Total								
			% Correct	% Hit	% Correct	% Hit	% Correct	% Hit	% Correct	% Hit	Correct Trials	Incorrect Trials	Omissions	Correct Latency	Total premature	% premature	Total trial #
Meta-analysis:																	
All subjects		106	57.4±2.2	34.6±1.9	72.0±1.5	56.3±1.9	73.7±1.3	63.0±1.8	67.7±1.3	51.3±1.6	42.2±2.4	18.1±1.2	20.4±1.5	0.60±0.03	5.8±0.6	5.2±0.5	107.4±2.6
"Low" Performers		35	41.9±4.3	13.7±1.2	66.5±3.1	38.0±2.8	70.5±3.1	50.9±3.3	59.6±2.5	34.2±2.1	29.2±3.3	15.5±1.7	33.2±2.7	0.70±0.07	3.0±0.08	3.5±1.0	90.0±6.1
“High” Performers		35	75.2±1.8 *	57.7±1.5*	80.2±1.7 *	72.8±1.8 *	78.9±2.1 *	73.9±2.3 *	78.1±1.5 *	68.1±1.4 *	55.6±4.6 *	16.2±1.8	10.9±1.3 *	0.48±0.02 *	7.7±0.9 *	6.4±0.8 *	120±0 *
Drug studies:																	
Vehicle		30	54.3±3.3	35.9±3.7	69.2±2.7	56.5±3.5	72.5±2.8	63.7±2.6	65.3±2.1	52.0±2.7	53.5±3.8	25.2±2.1	22.6±2.9	0.69±0.05	7.2±1.2	6.2±0.9	108.6±4.5
d-Amphetamine	0.03	30	59.9±3.1	39.6±3.4	72.9±2.0	60.5±2.7	74.0±2.1	64.1±2.7	68.9±1.8	54.7±2.5	57.7±3.5	24.2±1.5	22.6±2.6	0.63±0.04	8.5±1.5	7.3±1.2	113.0±2.9
d-Amphetamine	0.1	30	61.6±2.4	43.1±2.8 *	69.4±2.0	59.8±2.2	74.8±2.4	66.8±2.5	68.6±1.8	56.6±2.0	58.9±2.9	26.5±1.8	18.9±2.1	0.60±0.04	9.6±1.5	8.2±1.3	113.9±2.7
d-Amphetamine	0.3	30	65.7±2.3 *	53.1±3.0 *	74.9±2.3	67.5±2.6 *	75.2±2.8	68.6±3.0	72.0±1.7 *	63.1±2.2 *	61.1±3.4 *	23.3±1.8	10.9±1.8 *	0.51±0.02 *	16.6±2.2	14.5±1.9	111.8±4.2
Vehicle		23	61.9±3.9	43.2±4.4	70.2±2.3	58.6±3.6	68.9±2.7	59.3±3.3	67.0±2.6	53.7±3.4	60.1±3.9	27.2±1.8	24.4±3.4	0.72±0.08	5.9±1.0	5.0±0.8	117.7±1.5
Methylphenidate	1	23	58.±4.2	43.0±4.6	66.5±3.2	57.2±3.8	66.0±2.9	58.7±3.0	63.5±2.7	53.0±3.3	56.8±4.1	30.0±2.5	19.4±3.6	0.57±0.03 *	8.7±1.7	7.4±1.4	115.0±4.0
Methylphenidate	3	23	60.5±3.5	49.2±3.7	71.8±2.4	64.3±2.4	69.9±2.5	64.6±2.6	67.4±2.1	59.4±2.3	62.1±3.2	29.1±2.0	14.0±2.2 *	0.56±0.03 *	14.2±2.7 *	11.9±2.3 *	119.4±0.6
Methylphenidate	6	23	69.9±2.3	58.5±2.9 *	71.2±2.4	66.4±2.7 *	72.6±2.5	67.5±2.9 *	71.3±1.8	64.1±2.2 *	64.6±3.0	25.3±1.5	11.1±1.6 *	0.51±0.02 *	19.2±2.7 *	16.0±2.2 *	120±0
Vehicle		20	68.5±4.3	44.4±3.4	76.4±3.1	68.8±3.5	77.5±2.8	70.4±3.4	74.1±2.7	61.2±3.0	66.9±4.4	22.5±2.5	19.7±2.0	0.66±0.07	6.7±1.3	5.8±1.1	115.7±3.8
Nicotine	0.05	20	66.2±4.9	47.3±4.5	76.2±3.6	67.1±3.9	75.7±2.8	70.2±3.3	72.7±3.4	61.5±3.5	66.2±4.7	22.3±2.2	18.5±2.3	0.58±0.05 *	10.5±1.9	8.8±1.6	117.3±2.7
Nicotine	0.1	20	66.3±4.6	52.9±4.4	78.3±2.9	71.9±3.4	77.2±2.7	72.4±3.2	73.9±2.9	65.7±3.3	71.2±4.2	23.8±2.3	13.6±2.0 *	0.54±0.04 *	10.4±1.6	8.7±1.3	118.9±1.1
Nicotine	0.2	20	69.2±3.8	57.8±4.1 *	82.3±2.9	77.2±3.3 *	81.0±2.6	77.2±3.1 *	77.5±2.8	70.7±3.1 *	72.4±4.4	20.3±2.4	10.4±2.1 *	0.53±0.03 *	17.0±2.9 *	14.1±2.4 *	120±0
Vehicle		38	58.3±2.9	30.7±2.6	72.1±2.4	53.7±3.0	73.7±2.2	59.7±3.0	68.0±2.0	48.0±2.6	50.7±3.5	21.3±1.7	31.5±2.9	0.73±0.04	5.5±1.2	4.7±1.0	109.0±3.8
Atomoxetine	0.1	38	51.0±4.1	21.0±2.4 *	68.6±3.5	44.6±3.0 *	70.8±2.8	53.2±2.8 *	63.5±2.4	39.6±2.2 *	37.1±3.3 *	16.9±1.8 *	32.7±2.6	0.78±0.05	4.5±0.9	4.1±0.8	91.2±5.8 *
Atomoxetine	0.5	38	43.7±5.6	14.5±2.1 *	69.5±3.5	33.9±3.5 *	73.7±3.9	45.6±4.0 *	62.3±2.8	31.3±2.8 *	22.7±3.6 *	9.7±1.6 *	28.0±2.8	0.94±0.10	1.7±0.4 *	2.5±0.7 *	62.1±6.7 *
Atomoxetine	1	38	39.4±6.0 *	9.0±1.4 *	65.1±5.3	29.7±3.3 *	68.9±4.4	40.6±3.4 *	57.8±3.7	26.4±2.4 *	17.1±2.6 *	7.8±1.3 *	32.7±3.5	0.83±0.06	1.3±0.4 *	1.9±0.5 *	58.8±6.0 *

Data is presented as means ± SEM for all rats (N=106) tested under the sITI schedule, and for the “Low” and “High” performers (N=35) based on ranking to % hit at the 2-s ITI. Also shown are the performance scores following AMP (0.03–0.3 mg/kg), MPH (1–6 mg/kg), NIC (0.05–0.2 mg/kg) and ATX (0.1–1 mg/kg) pretreatment. *P<0.05 vs. “Low” performers (subgroup analysis), or vehicle pretreatment (drug studies).

A feature of the sITI task was that it identified rats with mixed performance. Notably this was most apparent at the most challenging test condition, i.e., 2-s ITI, with some rats making low levels of correct responses (termed “low performers”) and some rats making considerably higher levels of correct responses under same condition (termed “high performers”). Since differences in correct responses between these groups was due to both incorrect responses and missed responses (i.e. omissions), % hit was used as the selection measure and primary measure of accuracy in the sITI task in this subgroup analysis. Highly significant performance group differences were recorded for measures % hit (F1,68 = 181.5; P < 0.001; η_p_^2^ = 0.73), % correct (F1,68 = 39.6; P < 0.001; η_p_^2^ = 0.37), omissions (F1,68 = 68.4; P < 0.001; η_p_^2^ = 0.50), and correct latency (F1,68 = 12.3; P < 0.001; η_p_^2^ = 0.16) reflecting a strong association between each measure (see **Figure 1D**). “High performers” were characterized as showing higher accuracy, faster response speed and fewer omissions compared to their “low” counterparts. Despite the low overall level of premature responses, these also cosegregated with “high” and “low” performers (F1,68 = 14.5; P < 0.001; η_p_^2^ = 0.18), with “high performers” making more premature responses (total premature responses: 3.0 ± 0.8 vs. 7.7 ± 0.9).

Importantly, the performance of “high” and “low” performers remained consistent over repeated testing. This was demonstrated by two experiments. Firstly, a total of 89 rats were run for two consecutive sITI sessions, and a significant correlation in % hit (2-s ITI) was recorded (correlation=0.80; P < 0.01; see **Figure 1C**). Secondly, a cohort of 24 rats was subjected to repeated challenge sessions, presented at 3–4 day intervals. Restricting the analysis to challenge sessions 2–5, on the principal measures of % hit or % correct, there was no main effect of cycle (F3,69 ≤ 1.2, NS, η_p_^2^ ≤ 0.05) or cycle × ITI interaction (F6,138 ≤ 1.4, NS, η_p_^2^ ≤ 0.06) (see **Figure 1B**). Since the first cycle did produce a modest shift in performance, all drug studies under this schedule were conducted with the rats given an initial challenge exposure in the absence of drug treatment.

##### Characterization of AMP, MPH, NIC, ATX on sITI Task Performance

###### d-Amphetamine

**Figure 2:**
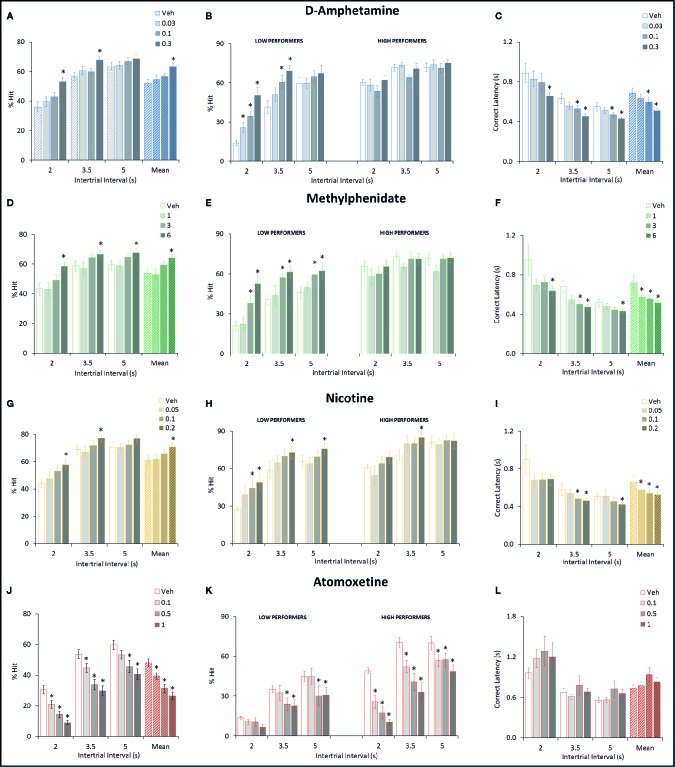
Effect of amphetamine (AMP), methylphenidate (MPH), nicotine (NIC), and atomoxetine (ATX) on attentional performance measured under the short intertrial interval (sITI) five-choice serial reaction time task (5-CSRTT) schedule. Performance accuracy (measured as % hit) of rats treated with AMP (0.03–0.3 mg/kg) **(A**–**C)**, MPH (1–6 mg/kg) **(D**–**F)**, NIC (0.05–0.2 mg/kg) **(G**–**I)**, and ATX (0.1–1 mg/kg) **(J**–**L)** tested under the sITI (2–5 s ITI, 0.3 s SD) 5-CSRTT schedule. For each drug three datasets are shown. (1) % hit at each ITI (sITI schedule) and also mean % hit collapsed across ITI **(A, D, G, J)**. (2) A summary of the “Low” and “High” performing tertile groups under each ITI level **(B, E, H, K)**. Tertile groups were selected based on performance under vehicle control (see methods for selection criteria). (3) Correct latency at each level of ITI and mean latency collapsed across ITI **(C, F, I, K)**. % hit used as primary measure of accuracy due to the relationship between omissions and task difficulty in each task schedule. Data presented as mean ± SEM. *P < 0.05 vs. vehicle pretreatment (LSD test following significant ANOVA).

AMP was tested at doses 0.03–0.3 mg/kg in a total N=30 rats. Main effects of AMP on measures of % hit (F3,87 = 8.6, P < 0.001; η_p_^2^ = 0.22), % correct (F3,87 = 5.4, P < 0.01; η_p_^2^ = 0.16), correct latency (F3,87 = 4.9, P < 0.01; η_p_^2^ = 0.15), and omissions (F3,87 = 10.0, P < 0.001; η_p_^2^ = 0.26), reflected AMP dose dependently improved performance by increasing accuracy, reducing missed trials and increasing response speed. A significant treatment × ITI interaction for most of these measures reflected that the effects of AMP were most evident under conditions of the highest challenge. Premature responses were also increased by AMP treatment (F3,87 = 15.2, P < 0.001; η_p_^2^ = 0.34). However, although significant, in numerical terms the increase in premature responses was relatively small (Veh: 7.2 ± 1.2, AMP: 0.3 mg/kg: 16.6 ± 2.2). See **Table 1** and **Figures 2A–C**. Subdividing the rats (N=30) into “low” and “high” performance (33:33 split, N=10 per group) based on % hit measure at 2-s ITI (vehicle pretreatment) revealed a significant performance level × treatment interaction of AMP on performance measures % correct (F3,54 = 3.6; P=0.01; η_p_^2^ = 0.17), % hit (F3,54 = 12.1, P < 0.001; η_p_^2^ = 0.40), omissions (F3,54 = 10.9, P < 0.001, η_p_^2^ = 0.38), which reflected that the most marked proattentive effects of AMP were in the “low” performers. See **Figure 2B**.

###### Methylphenidate

MPH was tested at doses 1–6 mg/kg in a total N=23 rats. Main effects of MPH on measures % hit (F3,66 = 8.4; P < 0.001; η_p_^2^ = 0.28), % correct (F3,66 = 4.2; P < 0.01; η_p_^2^ = 0.16), correct latency (F3,66 = 4.7; P < 0.01; η_p_^2^ = 0.18), omissions (F3,66 = 10.9; P < 0.001; η_p_^2^ = 0.33), reflected MPH dose dependently improved performance by increasing accuracy, reducing missed trials and increasing response speed. Although no significant treatment × ITI interaction was recorded for any of these measures, the effects of MPH seemed most evident under conditions of highest challenge. Premature responses were also increased by MPH treatment (F3,66 = 11.0; P < 0.001; η_p_^2^ = 0.33). Subdividing the rats (N=23) into “low” and “high” performance (33:33 split, N=8 per group) based on % hit measure at 2-s ITI (vehicle pretreatment) revealed even greater effects of MPH on performance. A significant performance level × treatment interaction of MPH on measures % hit (F3,42 = 5.7, P < 0.01; η_p_^2^ = 0.29), omissions (F3,42 = 7.2, P < 0.001; η_p_^2^ = 0.34) reflected that the most marked proattentive effects of MPH were in the “low” performers. % correct was of marginal significance (F3,42 = 2.3; P=0.09; η_p_^2^ = 0.14). Thus, the effects of MPH were qualitatively similar to AMP. See **Figures 2D–F** and **Table 1**.

###### Nicotine

NIC was tested at doses 0.05–0.2 mg/kg in a total N=20 rats. Main effects of NIC on measures % hit (F3,57 = 6.4; P < 0.001; η_p_^2^ = 0.25), correct latency (F3,57 = 7.0; P < 0.001; η_p_^2^ = 0.26), omissions (F3,57 = 7.4; P < 0.001; η_p_^2^ = 0.28), reflected NIC dose dependently improved performance by increasing hit rate, reducing missed trials and increasing response speed, although accuracy measured as % correct missed significance (F3,57 = 1.8; NS; η_p_^2^ = 0.09). Although no significant treatment × ITI interaction was recorded for any of these measures, the effects of NIC seemed most evident under conditions of highest challenge. Premature responses were also increased by NIC treatment (F3,57 = 9.7; P < 0.001; η_p_^2^ = 0.34). Subdividing the rats (N=20) into “low” and “high” performance (33:33 split, N=7 per group) based on % hit measure at 2-s ITI (vehicle pretreatment) revealed that the proattentive effects of nicotine seemed evident in both performance groups. Thus there was no significant performance level × treatment interaction of NIC on measures % hit (F3,36 = 1.6; NS; η_p_^2^ = 0.12), omissions (F3,36 = 1.4; NS; η_p_^2^ = 0.10) reflecting nicotine improvements in both measures were evident in both low and high performers. In this regard the effects of NIC were distinct to both AMP and MPH which improved performance essentially in the poor performer group only. See **Figures 2G–I** and **Table 1**.

###### Atomoxetine

ATX was tested at doses 0.1–1 mg/kg in a total N=38 rats. Main effects of ATX on measures % hit (F3,111 = 27.8, P < 0.001; η_p_^2^ = 0.43) and % correct (F3,111 = 3.7, P=0.01; η_p_^2^ = 0.09), reflected ATX dose dependently affected performance by reducing accuracy (both % correct and % hit), although no effect on omissions (F3,111 = 0.8, NS; η_p_^2^ = 0.02) or correct latency (F3,111 = 1.1, NS; η_p_^2^ = 0.07) was noted. Premature responses were significantly reduced by ATX (F3,111 = 10.4, P < 0.01; η_p_^2^ = 0.22), even after correcting for trial number that was reduced by atomoxetine (see **Figures 2J–L** and **Table 1**). Subdividing the rats (N=38) into “low” and “high” performance (33:33 split, N=13 per group) based on % hit measure at 2-s ITI (vehicle pretreatment) revealed even greater effects of ATX on performance. A significant performance level × treatment interaction of ATX on performance measures % correct (F3,72 = 2.9; P < 0.05; η_p_^2^ = 0.11), % hit (F3,72 = 4.9, P < 0.01; η_p_^2^ = 0.17), omissions (F3,72 = 11.1, P < 0.001; η_p_^2^ = 0.32) reflected that the most marked negative effects of ATX were in the “high performers” (see **Figure 2K**). The profile of ATX contrasted with AMP, MPH, and NIC on the sITI schedule in that performance was detrimentally affected following atomoxetine pretreatment.

#### sSD - Multiple Short Stimulus Duration

##### Characterization of Performance Under the sSD Task

**Figure 3:**
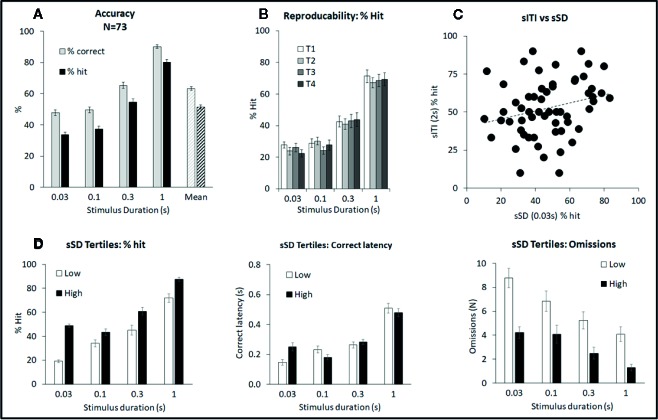
Summary of performance of adult male Long Evans rats in the short stimulus duration (sSD) five-choice schedule. Summary of performance of adult male Long Evans rats in the variable short stimulus duration (sSD) five-choice schedule. **(A)** Meta-analysis of 73 rats tested under sSD schedule—comparison of performance for accuracy (% correct and % hit) at each SD, and overall % accuracy collapsed across intertrial interval (ITI) (Mean). **(B)** Reproducibility of performance (% hit) following repeated exposures to test challenge (T1–T4). **(C)** Lack of correlation between individual rats tested under both the sSD and short ITI (sITI) task. Data is shown for the % hit measure at 2 s ITI and 0.03 s SD under both schedules. Rats were tested twice under each schedule over a 2-week period and mean performance was compared. The lack of correlation (Pearson correlation=0.25) indicates that performance level in one schedule, does not predict performance level in the other schedule. **(D)** Ranking rats according to % hit score under the 0.03 s SD. Low tertile group (N=24; “Low”) and high tertile group (N=24; “High”). In addition to higher accuracy, the “High” performer group had fewer omissions compared to the “Low” performer subgroup, however overall response speed (i.e. correct latency) was not related to performance.

The multiple SD test condition was designed to challenge the test subjects by presenting stimuli at shorter durations to reduce their discriminability, i.e. standard: SD=0.75 s; multiple SD=0.03, 0.1, 0.3, 1 s. All other parameters, including ITI (fixed 5 s) remained equivalent to standard conditions. A total of 73 rats were run in this task and a meta-analysis of data from all rats is presented in **Figure 3** and **Table 2**. Performance of rats in this test variant demonstrated the challenge of shortening the SD with accuracy measured either as % correct (F3,216 = 177.1; P < 0.001; η_p_^2^ = 0.71) or % hit (F3,216 = 260.5, P < 0.001; η_p_^2^ = 0.78) each showing a reliable stepwise decline as the SD decreased from 1 s to 0.3 s to 0.1–0.03 s (see **Figure 3A**). A significant main effect of SD on correct latency (F3,216 = 112.4; P < 0.001; η_p_^2^ = 0.61) and omissions (F3,216 = 57.0; P < 0.001; η_p_^2^ = 0.44) reflected that both omissions and response speed increased at the shorter SD. Premature responses were not significantly affected by varying the stimulus duration (F3,216 = 2.6, NS; η_p_^2^ = 0.04) and were low in range, being approximately 5% of total trials. Therefore, in the context of these experiments premature responses were considered of low importance.

**Table 2:** Summary of baseline performance and effect of amphetamine (AMP), methylphenidate (MPH), nicotine (NIC), and atomoxetine (ATX) on performance of rats in the short stimulus duration (sSD) schedule.

	Dose (mg/kg)	N	0.03s SD		0.1s SD		0.3s SD		1s SD		Total								
			% Correct	% hit	% Correct	% hit	% Correct	% hit	% Correct	% hit	% Correct	% hit	Correct Trials	Incorrect Trials	Omissions	Correct Latency	Total premature	% premature	Total trial #
Meta-analysis:																			
All subjects		73	47.9±1.9	33.8±1.6	49.7±1.9	37.4±1.7	65.4±1.9	54.6±2.1	90.3±1.2	80.2±1.7	63.2±1.2	51.5±1.4	50.2±2.0	26.2±1.0	19.8±1.5	0.30±0.01	10.5±1.2	11.6±1.6	96.0±2.6
"Low" Performers		24	32.5±2.6	19.1±1.2	47.6±3.3	34.1±2.9	57.1±4.0	45.0±4.1	86.4±2.7	71.8±3.7	55.9±2.4	42.5±2.4	40.5±3.5	28.0±1.9	25.0±2.6	0.29±0.01	7.1±1.3	7.7±1.5	93.5±5.2
"High" Performers		24	59.7±2.0 *	48.9±1.4 *	52.2±3.2 *	52.2±3.2 *	67.4±3.1 *	60.8±3.2 *	92.2±1.4 *	87.6±1.7 *	67.9±1.7 *	60.1±1.6 *	61.3±2.8 *	28.1±1.9	12.1±1.7 *	0.30±0.01	13.5±2.3 *	14.5±2.7 *	101.4±3.4
Drug studies:																			
Vehicle		26	45.1±2.6	34.7±2.6	47.3±2.3	38.5±2.1	67.6±2.3	57.5±2.7	92.5±1.7	85.8±1.9	63.1±1.4	54.1±1.6	52.2±2.8	29.3±2.0	14.5±2.2	0.29±0.01	14.5±2.0	15.4±2.4	96.0±4.1
d-Amphetamine	0.03	26	47.4±3.0	36.4±2.8	48.2±2.3	37.8±2.1	67.8±2.9	57.7±2.9	88.3±1.6	80.6±2.3	62.9±1.7	53.1±1.8	49.7±2.8	28.0±2.3	15.7±2.3	0.27±0.01	13.8±2.1	15.2±2.6	93.4±4.3
d-Amphetamine	0.1	26	42.4±2.8	32.1±2.4	48.6±2.8	38.6±3.2	69.5±3.4	56.6±3.8	90.4±1.4	80.5±2.4	62.7±1.7	51.9±2.4	47.0±3.3	26.0±2.1	15.0±2.3	0.27±0.02	14.4±2.6	16.3±3.2	88.0±4.7
d-Amphetamine	0.3	26	41.6±3.1	34.2±2.4	48.2±2.9	42.4±2.7	66.9±2.9	59.7±2.9	89.8±1.9	84.4±2.5	61.6±1.8	55.2±1.6	47.7±2.8	28.2±2.0	9.9±1.4 *	0.24±0.02 *	27.1±3.6 *	35.4±6.3 *	85.8±3.7
Vehicle		17	45.2±3.8	32.8±2.7	43.5±4.4	34.4±4.3	64.8±4.4	55.8±4.6	86.4±3.3	78.2±4.0	60.0±2.8	50.3±3.1	43.7±5.1	27.4±3.5	14.0±3.0	0.30±0.02	11.1±3.3	13.9±5.1	88.1±5.9
Methylphenidate	1	17	44.0±3.9	31.2±3.4	48.7±3.1	37.0±2.9	69.2±4.2	56.5±5.1	91.1±2.2	79.2±4.1	63.2±2.5	51.0±3.4	48.5±6.7	24.5±3.5	15.9±2.2	0.32±0.03	6.4±1.5	6.8±1.4	86.1±7.2
Methylphenidate	3	17	43.3±2.6	31.7±2.8	47.7±3.4	33.8±3.0	67.0±4.1	56.0±4.5	90.9±2.0	80.0±3.1	62.2±1.9	50.4±2.5	43.3±6.6	24.4±3.0	15.9±2.3	0.30±0.02	7.8±1.7	8.8±2.1	90.5±5.9
Methylphenidate	6	17	50.5±3.7	37.4±3.1	49.9±3.9	38.5±3.7	66.9±4.2	56.6±4.6	89.0±2.4	81.3±3.5	64.1±2.6	53.5±3.1	50.3±5.7	26.8±3.6	14.5±2.6	0.29±0.02	9.5±2.2	10.7±2.7	92.6±5.4
Vehicle		24	44.2±2.9	33.5±2.5	50.2±2.8	37.1±2.7	63.5±2.9	53.5±3.2	89.9±1.7	80.3±2.4	62.0±1.7	51.1±1.9	51.0±2.8	29.5±1.6	19.5±2.2	0.29±0.02	14.4±2.9	16.1±3.9	99.9±3.7
Nicotine	0.05	24	44.1±2.5	36.0±2.5	55.4±2.5	44.8±2.6	70.3±3.4	62.2±3.0	90.8±2.2	82.5±2.4	65.1±1.6	56.4±1.8	53.0±2.7	27.5±1.1	13.7±1.5	0.24±0.01 *	19.7±2.8	22.4±3.7	94.2±3.7
Nicotine	0.1	24	50.7±2.4	43.2±2.6	52.7±2.3	46.5±2.2	73.6±2.3	67.9±2.7	90.1±1.8	87.2±2.1	66.8±1.6 *	61.2±1.9 *	56.2±2.6	26.2±1.4	9.2±1.6 *	0.23±0.01 *	28.5±3.4 *	36.0±5.9 *	91.5±3.4
Nicotine	0.2	24	48.3±3.2	40.8±2.8 *	56.4±2.3	48.3±2.8 *	70.6±2.2 *	65.6±2.6 *	89.6±2.2	84.8±2.9	66.2±1.6 *	59.9±2.0 *	53.3±3.0	24.9±1.2 *	9.4±1.2 *	0.22±0.01 *	33.0±3.3 *	42.7±5.8 *	87.5±3.4
Vehicle		12	46.8±5.0	30.0±3.9	50.0±3.3	35.4±3.1	66.9±5.3	55.6±5.6	87.7±2.7	74.0±5.0	62.9±3.1	48.8±3.7	45.8±4.4	24.3±2.3	22.7±3.9	0.28±0.01	9.1±1.9	9.8±2.1	92.8±5.4
Atomoxetine	0.5	12	53.8±4.7	30.5±4.9	46.5±4.9	30.5±4.5	62.8±8.9	42.8±7.3	94.6±2.1	69.7±6.0	64.4±3.4	43.4±5.1	35.9±7.3 *	16.7±2.7 *	25.3±4.4	0.29±0.02	4.7±1.6 *	5.3±1.6 *	77.9±8.3
Atomoxetine	1	12	47.7±11.2	26.1±6.7	55.3±9.6	25.5±4.0	62.5±10.0	38.8±7.9	89.3±2.9	56.9±7.7	63.4±5.4	36.8±5.9	29.6±7.2 *	16.0±4.3 *	29.0±5.1	0.25±0.03	4.2±1.5 *	4.8±1.4 *	74.6±9.8

Data is presented as means ± SEM for all rats (N=73) tested under the sSD schedule, and for the “Low” and “High” performers (N=24) based on ranking to % hit at the 0.03 s SD. Also shown are the performance scores following AMP (0.03–0.3 mg/kg), MPH (1–6 mg/kg), NIC (0.05–0.2 mg/kg), and ATX (0.1–1 mg/kg) pretreatment. * P < 0.05 vs. “Low” performers (subgroup analysis), or vehicle pretreatment (drug studies).

Ranking the rats based on performance measure of % hit at the 0.03-s SD, i.e. the most extreme SD challenge resulted in “high” and “low” performers based on their respective tertiles (N=24 rats per tertile). Data for % hit, correct latency and omissions are shown in **Figure 3D**. Cosegregating with the % hit measure was omissions which were higher in the “low” performer group (F1,46 = 17.3; P < 0.001; η_p_^2^ = 0.27). Interestingly there was no overall difference in correct latency between these groups (F1,46 = 0.24; η_p_^2^ = 0.01) although a significant tertile × SD interaction was found (F3,138 = 5.1; P=0.002; η_p_^2^ = 0.10). This reflected that “Low” performers were faster under the 0.03-s SD, yet slower under the 0.1-s SD relative to their “High” counterparts.

A further experiment evaluated the effect of repeated test sessions on performance in a cohort of 24 rats. Single weekly challenges revealed that over 4 weeks all key performance measures remained stable—thus there was no main effect of week on % correct (F3,63 = 1.0, NS; η_p_^2^ ≤ 0.05), % hit (F3,63 = 0.3, NS; η_p_^2^ ≤ 0.01) (**Figure 3B**). No week × SD interaction was evident for % hit (F9,189 = 1.0, NS; η_p_^2^ ≤ 0.05) although % correct narrowly missed significance (F9,189 = 1.9, P=0.06; η_p_^2^ ≤ 0.08) likely reflecting a modest trend to improvement at the 0.3-s SD which by week 2–4 appeared to stabilize. Since the first cycle did produce a modest shift in performance, all drug studies under this schedule were conducted with the rats given an initial challenge exposure in the absence of drug treatment.

A final analysis was conducted between rat’s performance in the sSD and sITI schedules. For this experiment a cohort of rats (N=77) were run twice under each schedule and the mean % hit measure under each was determined. Correlational analysis of % hit under the most extreme challenge for each schedule (i.e., 2-s ITI sITI; 0.03-s SD sSD) failed to identify a significant correlation (correlation=0.25; NS, see **Figure 3C**) indicating that performance level in one task did not predict performance in the second task.

##### Characterization of AMP, MPH, NIC, ATX on Task Performance

###### d-Amphetamine

**Figure 4:**
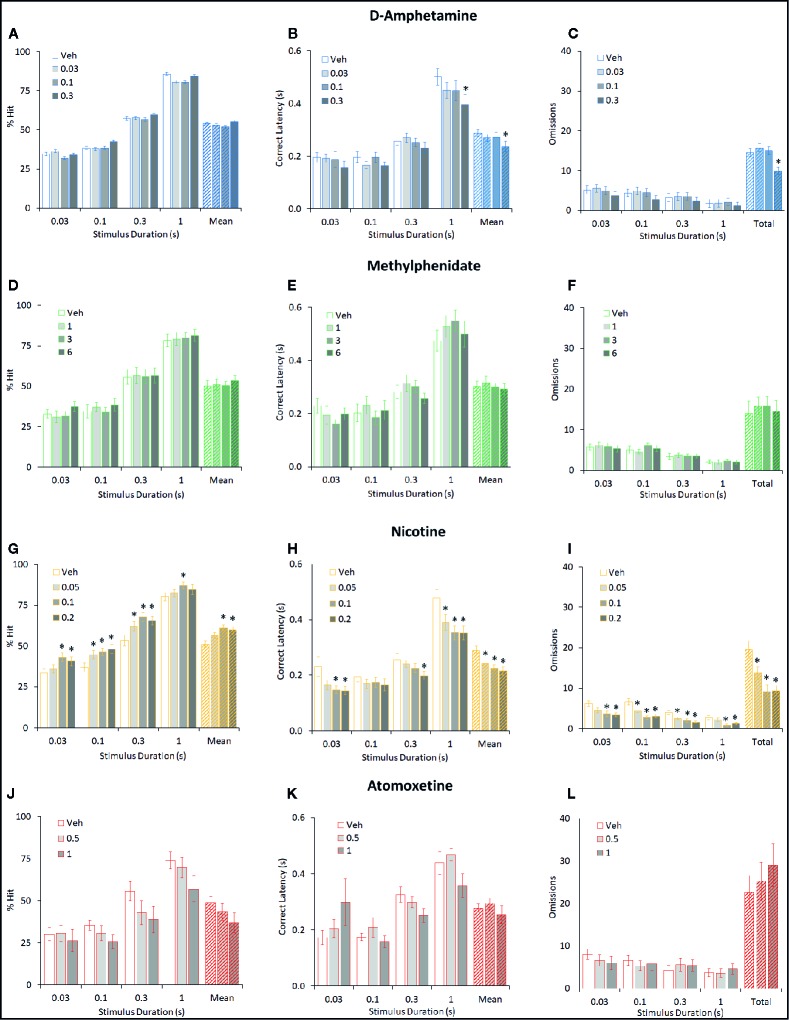
Effect of amphetamine (AMP), methylphenidate (MPH), nicotine (NIC), and atomoxetine (ATX) on attentional performance measured under the short stimulus duration (sSD) five-choice serial reaction time task (5-CSRTT) schedule. Performance accuracy (measured as % hit) of rats treated with AMP (0.03–0.3 mg/kg) **(A–C)**, MPH (1–6 mg/kg) **(D–F)**, NIC (0.05–0.2 mg/kg) **(G–I)**, and ATX (0.5–1 mg/kg) **(J–L)** tested under the multiple short stimulus duration (0.03–1 s SD; 5 s ITI; sSD) five-choice serial reaction time task schedule. For each drug three datasets are shown. (1) % hit at each level of SD and also mean % hit collapsed across all SD **(A, D, G, J)**. (2) Correct latency recorded at each level of SD and mean latency collapsed across all SD **(B, E, H, K)**. (3) Omissions recorded at each level of SD and omissions totalled across all SD **(C, F, I, K)**. % hit used as primary measure of accuracy due to the relationship between omissions and task difficulty in each task schedule. Data presented as mean ± SEM. *P < 0.05 vs. vehicle pretreatment (LSD test following significant ANOVA).

AMP (0.03–0.3 mg/kg IP; N=26 rats) did not improve overall accuracy measured either as % correct (63% to 61%; F3,75 = 0.5, NS, η_p_^2^ = 0.01) or % hit (54% to 55%; F3,75 = 0.9, NS, η_p_^2^ = 0.03). Omissions were also unaffected by amphetamine (F3,75 = 2.5, P=0.06, η_p_^2^ = 0.09) although there was a trend for the highest dose to reduce this measure. A main effect of AMP on premature responses (F3,75 = 7.3, P < 0.001, η_p_^2^ = 0.23) and correct latency (F3,75 = 3.0, P < 0.05, η_p_^2^ = 0.11) was recorded, reflecting the 0.3 mg/kg dose increasing premature responding and speed of responding compared to vehicle. See **Figures 4A–C** and **Table 2**. Subdividing the rats (N=26) into “low” and “high” performance (33:33 split, N=9 per group) based on % hit measure at 2-s ITI (vehicle pretreatment) failed to reveal a significant performance level × treatment interaction for % correct (F3,48 = 0.5; NS; η_p_^2^ = 0.04) although the % hit measure met significance (F3,48 = 2.9; P=0.04; η_p_^2^ = 0.16). This interaction reflected a modest trend toward improvement in the low performance group (veh: 46.9% ± 2.1%, amp 0.3 mg/kg: 53.5% ± 2.7%) and decline in the high performance group (veh: 59.6% ± 2.0%, amp 0.3 mg/kg: 55.4% ± 2.9%).

###### Methylphenidate

Similarly, MPH (1–6 mg/kg IP; N=17 rats) did not significantly improve overall accuracy measured either as % correct (60% to 64%; F3,48 = 1.1, NS; η_p_^2^ = 0.06) or % hit (50% to 54%; F3,48 = 0.5, NS; η_p_^2^ = 0.03). With the exception of premature responses, there was no main effect of treatment or treatment × SD interaction on measures such as omissions, response speed, overall trials completed. A significant treatment × SD interaction on premature responses (F9,144 = 2.6, P < 0.01; η_p_^2^ = 0.14) reflected a modest decrease following MPH treatment at the short SD’s. See **Figures 4D–F** and **Table 2**. Subdividing the rats (N=17) into “low” and “high” performance (33:33 split, N=6 per group) based on % hit measure at 2-s ITI (vehicle pretreatment) failed to reveal a significant performance level × treatment interaction for measures of % correct (F3,30 = 1.5; NS; η_p_^2^ = 0.13) or % hit (F3,30 = 1.2; NS; η_p_^2^ = 0.10).

###### Nicotine

NIC (0.05–0.2 mg/kg SC; N=24 rats) improved overall accuracy measured both as % correct (62% ± 2% to 67% ± 2%; F3,69 = 3.2, P < 0.05; η_p_^2^ = 0.12) and % hit (51% ± 2% to 61% ± 2%; F3,69 = 7.9, P < 0.001; η_p_^2^ = 0.25). On both measures of % correct and % hit there was no significant treatment × SD interaction (F9,207 ≤ 1.3, NS; η_p_^2^ ≤ 0.05), reflecting that nicotine improved performance across all SD’s. Main effects of treatment on correct latency (F3,69 = 10.6, P < 0.001; η_p_^2^ = 0.32), omissions (F3,69 = 10.8, P < 0.001; η_p_^2^ = 0.32) revealed nicotine to increase speed of responding, i.e. faster to respond, and reduce omissions reflecting an overall performance improvement both in accuracy and speed of responding. Nicotine also increased premature responses (F3,69 = 14.7, P < 0.001; η_p_^2^ = 0.39). All effects were related to dose. See **Figures 4G–I** and **Table 2**.

A subgroup analysis was conducted on the effect of nicotine in the “High” and “Low” performer groups (N=8 per subgroup). The performance level × treatment interaction for % correct (F3,42 = 2.5; P=0.08; η_p_^2^ = 0.15), % hit (F3,42 = 2.8; P=0.05; η_p_^2^ = 0.17) and omission (F3,42 = 2.8; P=0.05; η_p_^2^ = 0.17) measures were of borderline significance and reflected a trend for the proattentive effect of nicotine to be most prominent in the “low” performing group. The effect of NIC on response speed was independent of performance level (F3,42 = 0.2; NS; η_p_^2^ = 0.01).

###### Atomoxetine

ATX (0.5–1 mg/kg; N=12 rats) had no effect on accuracy measured either as % correct (F2,12 = 0.5, NS; η_p_^2^ = 0.07) or % hit (F2,12 = 1.6, NS; η_p_^2^ = 0.21). A main effect of atomoxetine on premature responses (F2,22 = 7.3, P < 0.01; η_p_^2^ = 0.40) but not correct latency (F2,22 = 0.2, NS; η_p_^2^ = 0.04), reflected that atomoxetine reduced premature responding without slowing response speed. Despite no main effect on accuracy, a proportion of subjects pretreated with ATX (2/12 at 0.5 mg/kg, and 5/12 at 1 mg/kg) did not complete any correct/incorrect trials under the 0.03-s to 0.3-s SD condition, resulting in missing data. See **Figures 4J–L** and **Table 2**. Due to the relatively low group size (N=4 per subgroup), no subgrouping analysis was run on this study cohort.

#### Long 10-s ITI – Low Rate of Stimulus Presentation

##### Characterization of Performance Under the Long 10-s ITI Task

**Figure 5:**
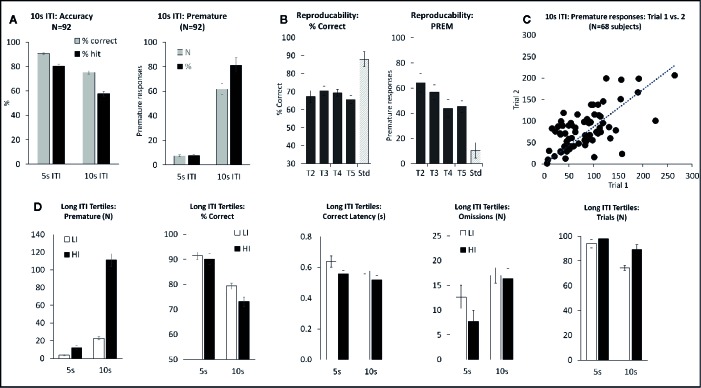
Summary of performance of adult male Long Evans rats in the 10-s intertrial interval (ITI) five-choice schedule. Summary of performance of adult male Long Evans rats in the 10-s ITI five-choice schedule. **(A)** Meta-analysis of 92 rats tested under this schedule – comparison of performance for accuracy (% correct and % hit) and premature responding (total # prematures and % prematures) between 5-s ITI (0.75-s SD) and 10-s ITI (0.3-s SD). **(B)** Reproducibility of performance following repeated exposures to test challenge (T2-T5). **(C)** Rats show a wide variation in terms of # premature responses emitted during a 10-s ITI session. These differences are reasonably consistent across repeated testing. **(D)** Ranking rats according to # premature responses—low tertile group (N=30) termed “Low impulsives” (LI) and high tertile group (N=30) termed “High impulsives” (HI). The HI group have higher premature responses under standard test conditions (5-s ITI) as well as under longer ITI, and also have faster response latencies. Accuracy similar at 5-s ITI between LI and HI, but lower in the HI group at 10-s ITI likely due to premature responses coincident with stimulus onset.

The long 10-s ITI test condition was designed to challenge the test subjects by presenting stimuli at long intervals and thus taxing the animal’s ability to wait before making a response. The stimulus duration was also reduced from 0.75 to 0.3 s for these sessions. All other parameters remained equivalent to standard conditions. The performance of a cohort of rats (N=92) was assessed in the long 10-s ITI schedule and compared to their performance under standard conditions (5-s ITI, 0.75-s SD, 100 trials) assessed 1–2 days before or after the test challenge. As predicted, premature responses were reliably increased by lengthening the ITI, measured either as absolute number or as a percentage of trial number (see **Figure 5A**). Accuracy measured as % correct was significantly reduced under the extended ITI condition (T(91)=12.5; P < 0.001). Speed of responding (measured as correct latency) was increased (i.e. faster) (T(91)=3.4; P=0.001), and omissions increased (T(91)=5.5; P < 0.001) under the 10-s ITI compared to 5-s ITI condition (see **Figures 5A–D**).

Similar to the other challenge tasks, across test subjects a variable performance was evident on the level of premature responses made under the 10-s ITI condition, with some rats making low levels of premature responses (termed “low impulsives” [LI]) and others making considerably higher levels of premature responses under the same condition (termed “high impulsives” [HI]). Segregating rats into LI and HI tertiles based on the number of premature responses made under the 10-s ITI (N=30 rats per tertile) revealed the HI rats to also make more premature responses under the 5-s ITI (5-s ITI: LI group 3.7 ± 0.8; HI group 12.0 ± 2.2; P < 0.001). HI rats were also faster to make a correct response (F1,58 = 4.3; P < 0.05; η_p_^2^ = 0.07) and initiate more trials (F1,58 = 5.4; P < 0.05; η_p_^2^ = 0.08). Accuracy measured as % correct narrowly missed significance (F1,58 = 3.4, P=0.07; η_p_^2^ = 0.05) with the HI rats showing slightly lower accuracy (see **Figure 5D**).

The performance of “high” and “low” impulsives appeared consistent over repeated testing based on outcomes from two control experiments. Firstly, a total of 68 rats were run for 2 consecutive long 10-s ITI sessions, and a significant correlation in overall premature responses was recorded (correlation=0.63; P < 0.01; see **Figure 5C**). Secondly, a cohort of 34 rats was subjected to repeated challenge sessions, presented at 7-day intervals. Restricting the analysis to challenge sessions 2–5, on the principal measures of number of premature responses and % correct, there was no main effect of cycle (premature: F3,99 = 1.4, NS; η_p_^2^ = 0.04) (% correct: F3,99 = 0.8, NS; η_p_^2^ = 0.02) (see **Figure 5B**). Since the first cycle did produce a modest shift in performance in both measures, all drug studies under this schedule were conducted with the rats given an initial challenge exposure in the absence of drug treatment.

##### Characterization of AMP, MPH, NIC, ATX on Task Performance

**Figure 6:**
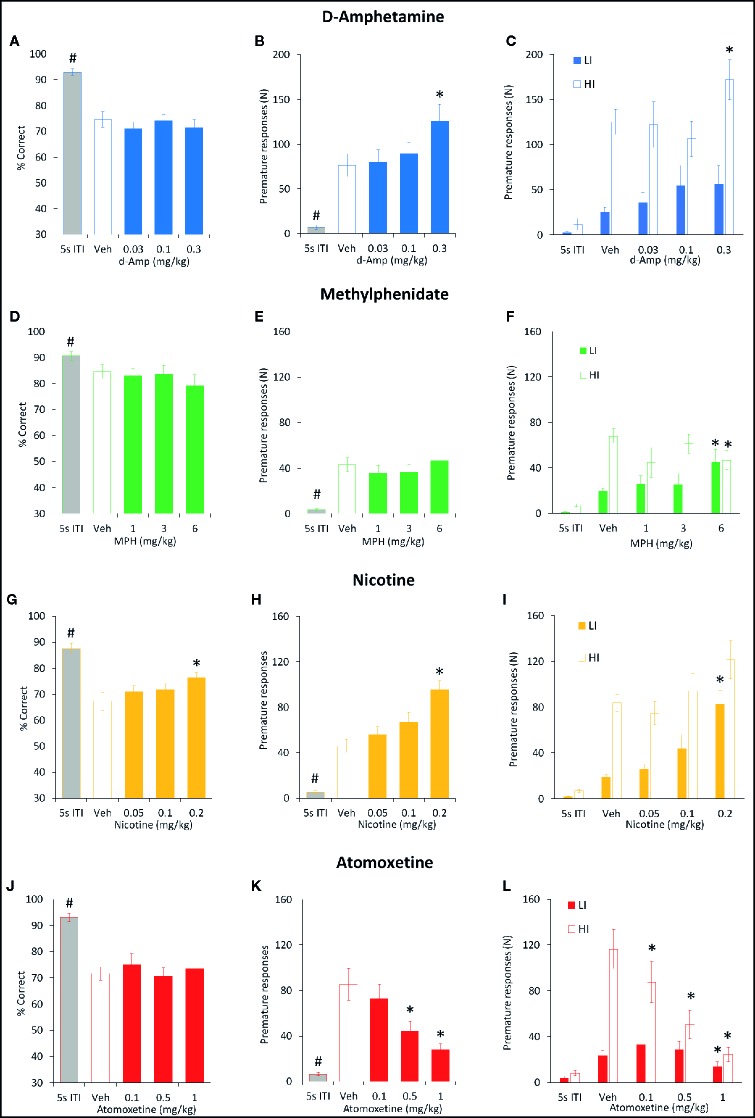
Effect of amphetamine (AMP), methylphenidate (MPH), nicotine (NIC), and atomoxetine (ATX) on attentional accuracy (% correct) and impulsive action measured under the long 10-s ITI 5-CSRTT schedule. Performance accuracy (measured as % correct) and premature responses of rats treated with AMP (0.03–0.3 mg/kg) **(A–C)** , MPH (1–6 mg/kg) **(D–F)**, NIC (0.05–0.2 mg/kg) **(G–I)**, and ATX (0.1–1 mg/kg) **(J–L)** tested under the long (10 s) ITI (0.3 s SD) five-choice serial reaction time task schedule. For each drug three datasets are shown. (1) % correct for all rats tested under 10 s ITI **(A, D, G, J)**. (2) Number of premature responses for all rats tested under 10 s ITI **(B, E, H, K)**. (3) Number of premature responses according to the “Low” impulsive (LI) and “High” impulsive (HI) tertile groups **(C, F, I, L)**. Tertile groups were selected based on performance under vehicle control (see methods for selection criteria). For comparative purpose the performance of rats under standard conditions (5 s ITI, 0.75 s SD) measured during the testing cycle is also included (5 s ITI). % correct was used as primary measure of accuracy. Data presented as mean ± SEM. * P < 0.05 vs. vehicle pretreatment (LSD test following significant ANOVA). ^#^P < 0.05 vs. Veh 10s ITI.

Consistent with the task characterization outcomes, in all the drug studies, increasing the ITI from 5 to 10 s, and reducing SD from 0.75 to 0.3 s resulted in reliable decreases in attentional accuracy and increases in premature responses. Subgrouping the rats according to premature responding under 10-s ITI, also confirmed the HI animals as having significantly higher premature responses at 5-s ITI, albeit at a much lower level (see **Figure 6**).

###### d-Amphetamine

The effect of AMP (0.03–0.3 mg/kg) on performance under the long 10-s ITI was evaluated in a total of 16 rats. The only main effect directly attributable to AMP was on premature responses (F4,60 = 19.9, P < 0.001; η_p_^2^ = 0.57) which were significantly increased at the 0.3 mg/kg dose. Other main effects reflected reduced accuracy (% correct, % hit) and increased omissions produced by the 10-s ITI schedule relative to baseline and these were unaffected by AMP. Subgrouping into LI and HI groups (N=6 per subgroup) identified the proimpulsive effect of AMP to be significant only in the HI group (treatment × subgroup interaction: F4,40 = 4.5; P < 0.01; η_p_^2^ = 0.31). See **Figures 6A–C** and **Table 3**.

**Table 3:** Summary of baseline performance and effect of amphetamine (AMP), methylphenidate (MPH), nicotine (NIC), and atomoxetine (ATX) on performance of rats in the 10sITI schedule.

	Dose (mg/kg)	N	ITI	% Correct	% Hit	Correct trials	Incorrect trials	Omissions	Correct latency	Total prematures	% Prematures	Total trial #
Meta-analysis:												
All subjects	-	92	5s	90.5 ± 0.8	80.4 ± 1.5	78.4 ± 2.0	7.7 ± 0.6	10.2 ± 1.1	0.61 ± 0.02	7.4 ± 0.9	7.7 ± 1.0	96.2 ± 1.4
	-	92	10s	75.2 ± 1.3 *	57.7 ± 2.0 *	50.2 ± 2.5 *	14.8 ± 0.9 *	17.3 ± 1.3 *	0.56 ± 0.01 *	61.9 ± 4.5 *	81.3 ± 6.0 *	82.2 ± 2.6 *
“Low” Impulsive (LI)	-	30	5s	91.4 ± 1.5	77.7 ± 3.4	75.0 ± 4.6	6.3 ± 1.0	12.7 ± 2.4	0.64 ± 0.04	3.7 ± 0.8	3.7 ± 0.8	94.0 ± 3.4
	-	30	10s	79.3 ± 2.2 *	57.3 ± 4.0 *	46.0 ± 4.8 *	11.3 ± 1.5 *	17.0 ± 2.3 *	0.58 ± 0.02 *	22.7 ± 1.9 *	38.9 ± 6.4 *	74.3 ± 5.1 *
“High” Impulsive (HI)	-	30	5s	90.1 ± 1.3	83.4 ± 2.1	81.9 ± 2.8	8.4 ± 1.0	7.7 ± 1.5	0.56 ± 0.02	12.0 ± 2.2	12.6 ± 2.2	98.0 ± 2.0
	-	30	10s	73.1 ± 1.8 *#	59.0 ± 3.1 *	54.6 ± 4.1 *#	18.4 ± 1.5 *#	16.3 ± 2.0 *	0.52 ± 0.03 *#	111.1 ± 7.2 *#	131.2 ± 9.2 *#	89.3 ± 3.8 *#
Drug studies:												
Baseline	-	16	5s	92.9 ± 1.2 *	84.1 ± 2.4 *	84.1 ± 2.4 *	6.3 ± 1.1 *	9.6 ± 2.0 *	0.59 ± 0.04	7.0 ± 2.5 *	7.0 ± 2.5 *	100 ± 0
Vehicle	-	16	10s	74.5 ± 3.0	56.2 ± 5.7	55.7 ± 5.9	18.9 ± 1.9	22.1 ± 5.0	0.56 ± 0.04	76.4 ± 12.5	78.5 ± 12.4	96.7 ± 2.3
d-Amphetamine	0.03	16	10s	70.9 ± 2.7	56.5 ± 5.2	55.0 ± 5.7	20.1 ± 1.8	19.9 ± 4.3	0.54 ± 0.03	79.8 ± 14.0	88.9 ± 17.9	95.0 ± 2.9
d-Amphetamine	0.1	16	10s	74.1 ± 2.5	61.8 ± 4.1	58.0 ± 5.5	19.5 ± 2.2	14.2 ± 2.7	0.55 ± 0.04	89.2 ± 13.0	119.8 ± 30.4	91.7 ± 5.2
d-Amphetamine	0.3	16	10s	71.4 ± 3.2	57.2 ± 5.0	55.1 ± 5.8	20.4 ± 2.1	17.8 ± 3.2	0.55 ± 0.03	125.8 ± 18.8 *	149.5 ± 27.7 *	93.3 ± 4.6
Baseline	-	16	5s	90.7 ± 1.6	77.2 ± 3.9	67.3 ± 6.9	6.6 ± 1.2	9.4 ± 1.7	0.68 ± 0.04	3.4 ± 1.0 *	4.8 ± 1.8 *	83.3 ± 6.6
Vehicle	-	16	10s	84.7 ± 2.6	69.4 ± 3.9	60.7 ± 6.1	10.8 ± 2.3	13.4 ± 1.9	0.63 ± 0.04	43.2 ± 6.0	50.2 ± 5.6	84.9 ± 5.8
Methylphenidate	1	16	10s	83.1 ± 2.6	61.9 ± 4.1	44.3 ± 6.3	8.9 ± 1.7	15.9 ± 2.0	0.69 ± 0.05	35.8 ± 6.9	50.4 ± 6.3	69.1 ± 7.5
Methylphenidate	3	16	10s	83.7 ± 3.4	56.8 ± 5.8	42.6 ± 7.4	6.8 ± 1.7	18.0 ± 2.6	0.62 ± 0.05	36.6 ± 6.9	52.2 ± 8.6	67.3 ± 6.6
Methylphenidate	6	16	10s	79.2 ± 4.3	60.4 ± 6.1	51.4 ± 6.8	10.3 ± 1.8	19.6 ± 3.2	0.67 ± 0.06	46.5 ± 7.4	56.3 ± 8.7	81.3 ± 5.8
Baseline	-	29	5s	87.5 ± 2.0 *	71.5 ± 3.8 *	69.8 ± 4.2 *	8.1 ± 1.0 *	16.0 ± 2.4	0.69 ± 0.05 *	5.5 ± 1.3 *	5.7 ± 1.4 *	93.6 ± 3.5 *
Vehicle	-	29	10s	67.4 ± 3.5	50.7 ± 4.1	43.0 ± 5.0	15.1 ± 1.7	18.4 ± 2.7	0.54 ± 0.04	46.1 ± 5.8	72.2 ± 10.9	76.5 ± 5.4
Nicotine	0.05	29	10s	70.9 ± 2.3	54.4 ± 3.8	46.4 ± 4.9	15.5 ± 1.6	15.6 ± 2.1	0.54 ± 0.03	56.0 ± 6.8	89.4 ± 17.2	77.5 ± 5.5
Nicotine	0.1	29	10s	71.8 ± 2.5	54.6 ± 4.5	49.6 ± 5.5	15.1 ± 1.8	16.3 ± 2.3	0.56 ± 0.03	66.9 ± 8.6 *	103.4 ± 20.5 *	81.0 ± 5.6
Nicotine	0.2	29	10s	76.3 ± 2.0 *	61.0 ± 4.0 *	56.8 ± 4.8 *	15.4 ± 1.6	16.1 ± 2.5	0.50 ± 0.03	95.4 ± 7.9 *	125.7 ± 22.4 *	88.3 ± 4.3
Baseline	-	28	5s	92.5 ± 1.0 *	85.1 ± 1.7 *	84.1 ± 2.2 *	6.8 ± 0.9 *	7.7 ± 1.2 *	0.62 ± 0.02 *	5.6 ± 1.0 *	5.6 ± 1.0 *	98.5 ± 1.2 *
Vehicle	-	28	10s	70.3 ± 3.1	53.2 ± 3.9	43.9 ± 5.0	14.4 ± 1.3	16.1 ± 2.0	0.52 ± 0.03	66.5 ± 9.8	96.5 ± 12.5	74.4 ± 5.1
Atomoxetine	0.1	28	10s	73.8 ± 3.1	56.3 ± 4.2	46.4 ± 5.5	13.3 ± 1.6	13.8 ± 2.0	0.55 ± 0.04	55.9 ± 8.5	94.3 ± 19.2	72.9 ± 6.0
Atomoxetine	0.5	28	10s	72.2 ± 2.5	48.0 ± 4.0	38.0 ± 5.2	12.9 ± 1.9	18.9 ± 2.4	0.53 ± 0.02	35.5 ± 5.7 *	55.8 ± 8.5 *	69.2 ± 6.5
Atomoxetine	1	28	10s	73.7 ± 2.9	42.9 ± 3.9 *	26.3 ± 4.6 *	10.1 ± 1.9 *	17.9 ± 2.5	0.55 ± 0.02	22.2 ± 3.5 *	46.0 ± 6.5 *	54.3 ± 6.1 *

Data is presented as means ± SEM for all rats (N=92) tested under the sITI schedule, and for the “Low” and “High” impulsives (N=30) based on ranking to the level of premature responding at the 10-s ITI. Also shown are the performance scores following AMP (0.03–0.3 mg/kg), MPH (1–6 mg/kg), NIC (0.05–0.2 mg/kg), and ATX (0.1–1 mg/kg) pretreatment. * P < 0.05 vs. respective subgroup at 5-s ITI (subgroup analysis), or vehicle pretreatment (drug studies). # P < 0.05 vs. “Low” impulsives at respective ITI.

###### Methylphenidate

MPH (1–6 mg/kg) had little effect on both accuracy and premature responding when all rats (N=16) were included in the analysis. All main effects reflected reduced accuracy (% correct, % hit) and increased omissions produced by the 10-s ITI schedule relative to baseline and these were unaffected by MPH. However, subgrouping into LI and HI groups (N=6 per subgroup) identified the effect of MPH on premature responses to be related to subgroup (treatment × subgroup interaction: F4,40 = 4.5; P < 0.01; η_p_^2^ = 0.31). Specifically, MPH reduced premature responses in the HI group, and increased this measure in the LI group. No other measures showed a significant MPH × subgroup interaction. See **Figures 6D–F** and **Table 3**.

###### Nicotine

NIC (0.05–0.2 mg/kg) was evaluated in a total of 29 rats, and main effects on both accuracy and premature responses were recorded. A main effect of group was recorded on % correct (F4,112 = 23.9, P < 0.001; η_p_^2^ = 0.46), in part reflected an increase in nicotine 0.2 mg/kg compared to vehicle pretreatment (Veh: 67.4% ± 3.5%, Nic 0.2 mg/kg: 76.3% ± 2.0%; P < 0.001), due to an increase in the number of correct trials (Veh: 43.0 ± 5.0, Nic 0.2 mg/kg: 56.8 ± 4.8; P < 0.01). A main effect of treatment on premature responding was also recorded (F4,112 = 39.2, P < 0.001; η_p_^2^ = 0.58), with both the 0.1 (P < 0.05) and 0.2 mg/kg (P < 0.001) doses increasing premature responding relative to vehicle pretreatment at the 10-s ITI. Subgrouping into LI and HI groups (N=9 per group) identified a trend to a treatment × subgroup interaction for % correct (F4, 64 = 2.3, P=0.06; η_p_^2^ = 0.13) and a significant treatment × subgroup interaction for premature responses (F4, 64 = 3.2, P=0.02; η_p_^2^ = 0.17). This reflected the effect of nicotine on both measures, i.e. increasing accuracy and premature responses, was most evident in the LI relative to the HI group. See **Figures 6G–I** and **Table 3**.

###### Atomoxetine

The effect of ATX (0.1–1 mg/kg) on a fixed long ITI procedure (10-s ITI, 0.3-s SD) was evaluated in a total of 28 rats. ATX treatment significantly reduced premature responding (F4,108 = 21.0, P < 0.001; η_p_^2^ = 0.43) with significant effects at 0.5 and 1 mg/kg dose. Although a main effect of group was recorded on % correct (F4,108 = 17.8; P < 0.001; η_p_^2^ = 0.41), ATX had no effect relative to vehicle control. However, a main effect of group on % hit (F4,108 = 35.5; P < 0.001; η_p_^2^ = 0.57) revealed the highest dose of ATX (1 mg/kg) to reduce this measure relative to vehicle pretreatment. Main group effects on omissions (F4,108 = 7.1; P < 0.001; η_p_^2^ = 0.21) and correct latency (F4,108 = 3.0; P < 0.05; η_p_^2^ = 0.10) only reflected differences between vehicle pretreatment under 5-s vs. 10-s ITI, and not ATX pretreatment. Subgrouping the rats into LI and HI groups (N=10 per subgroup) revealed a significant treatment × subgroup interaction for premature response (F4,72 = 9.3; P < 0.001; η_p_^2^ = 0.34) and omissions (F4,72 = 4.4; P < 0.01; η_p_^2^ = 0.20). This reflected that premature responding in the HI subgroup was most sensitive to ATX, with all doses decreasing this measure (see **Figure 6L**). ATX also increased omissions selectively in the HI subgroup. See **Figures 6J–L** and **Table 3**.

### Go/NoGo Task

For each experiment, on Go trials the performance of rats under baseline conditions and vehicle pretreatment was >95% correct, on NoGo trials performance was in the range 50%–60% correct. This difference in performance between the two trial types reflected that rats readily learned the “Go” task, but often required correction training to learn the “NoGo” trials (i.e. occasional forced “NoGo” trials were required during training) and never reached the performance level under “Go” trials. Response latencies averaged over the drug studies were 2.17 ± 0.08 s (correct latency) and 2.79 ± 0.24 s (incorrect latency).

#### d-Amphetamine

**Figure 7:**
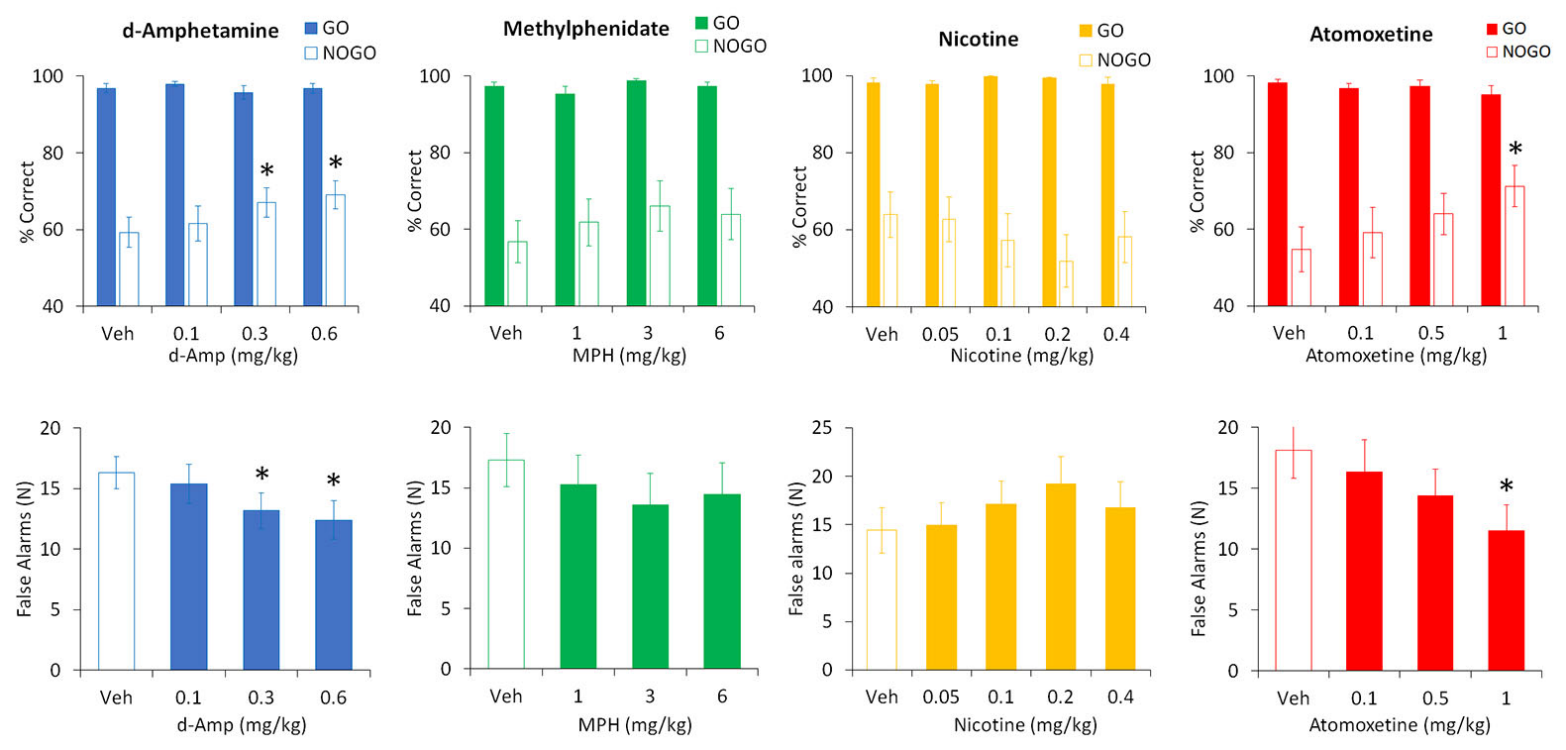
Effect of amphetamine (AMP), methylphenidate (MPH), nicotine (NIC), and atomoxetine (ATX) on performance in a symmetrically reinforced Go-NoGo task. Effect of AMP (0.1–0.6 mg/kg), MPH (1–6 mg/kg), NIC (0.05–0.4 mg/kg), and ATX (0.1-1 mg/kg) on performance in a symmetrically reinforced Go-NoGo task. N=12–20 Long Evans rats received each treatment in a balanced design (see Methods for details). The upper panels show the % correct responding under the Go or NoGo trials, the lower panels show the number of false alarms (incorrect responses made during the NoGo trials). * P < 0.05 vs. vehicle pretreatment (Dunnett’s test following significant ANOVA).

AMP was tested at doses 0.1–0.6 mg/kg (N=13 rats). Main effects of treatment were found on total % correct (F3,36 = 4.5, P < 0.01; η_p_^2^ = 0.27) and a two-way ANOVA revealed a treatment × trial-type interaction (F3,36 = 3.1, P < 0.05; η_p_^2^ = 0.20) reflecting AMP improved performance primarily under the “NoGo” trial condition. Performance improvement was due to AMP reducing false alarms (F3,36 = 4.7, P < 0.01; η_p_^2^ = 0.30). Post hoc tests identified significance at AMP doses of 0.3 and 0.6 mg/kg. There was no effect of AMP on either correct or incorrect latency (F3,36 ≤ 1.3, NS; η_p_^2^ ≤ 0.1). See **Figure 7**.

#### Methylphenidate

MPH was tested at doses of 1–6 mg/kg (N=14 rats). Despite showing a modest trend to improved accuracy by reducing false alarms, no significant main effect of treatment was found on total % correct (F3,39 = 2.2, P=0.1; η_p_^2^ = 0.14), trial type (F3,39 = 2.2, P=0.1; η_p_^2^ = 0.14), treatment × trial type (F3,39 = 1.4, NS; η_p_^2^ = 0.09), or false alarms (F3,39 = 1.7, NS; η_p_^2^ = 0.12). Similarly there was no effect of MPH on either correct or incorrect latency (F3,39 ≤ 0.9, NS; η_p_^2^ ≤ 0.07). See **Figure 7**.

#### Nicotine

Nicotine was tested at doses of 0.05–0.4 mg/kg (N=12 rats). No significant main effect of treatment was found on total % correct (F4,44 = 1.0, NS; η_p_^2^ = 0.08), and a two-way ANOVA failed to reveal a treatment × trial-type interaction (F4,44 = 1.5, NS; η_p_^2^ = 0.12). Similarly, there was no effect of nicotine on false alarms (F4,44 = 1.3, NS; η_p_^2^ = 0.11) or correct or incorrect latency (F4,44 ≤ 0.7, NS). See **Figure 7**.

#### Atomoxetine

ATX was tested at doses of 0.1–1 mg/kg (N=20 rats). Main effects of treatment were found on total % correct (F3,57 = 4.6, P < 0.01; η_p_^2^ = 0.20) and a two-way ANOVA revealed a treatment × trial-type interaction (F3,57 = 6.0, P < 0.01; η_p_^2^ = 0.24) reflecting ATX improved performance primarily under the “NoGo” trial condition. False alarms were also reduced by ATX (F3,57 = 6.1, P < 0.01; η_p_^2^ = 0.24). Post hoc tests identified significance at the ATX dose of 1 mg/kg. There was no effect of ATX on either correct or incorrect latency (F3,57 ≤ 1.5, NS; η_p_^2^ ≤ 0.07). See **Figure 7**.

### Progressive Ratio Task

#### d-Amphetamine

AMP (0.03–0.6 mg/kg) had a significant main effect of treatment on the number of lever presses (F4,28 = 9.3; P < 0.001; η_p_^2^ = 0.57), break point (F4,28 = 12.6; P < 0.001; η_p_^2^ = 0.64), and total session duration (F4,28 = 16.6; P < 0.001; η_p_^2^ = 0.70). On each measure d-amphetamine produced a dose related increase in responding, increasing the number of lever presses, the break point and consequently session duration. Significant differences relative to control were evident at 0.3–0.6 mg/kg doses. See **Table 4**.

**Table 4:** Summary of effects of amphetamine (AMP), methylphenidate (MPH), nicotine (NIC), and atomoxetine (ATX) on responding for food under a progressive ratio schedule of reinforcement.

		Break point	No. active lever press	Session duration (min)
**d-Amphetamine**	Vehicle	11.3 ± 0.7	303 ± 41	14.6 ± 1.7
	0.03 mg/kg	11.8 ± 1.0	336 ± 42	17.8 ± 2.6
	0.1 mg/kg	12.9 ± 1.0	473 ± 120	23.1 ± 3.7
	0.3 mg/kg	14.4 ± 1.1 **	693 ± 173	32.9 ± 3.4 **
	0.6 mg/kg	17.1 ± 1.4 **	1461 ± 362 **	45.8 ± 5.3 **
**Methylphenidate**	Vehicle	11.6 ± 0.7	307 ± 38	17.3 ± 4.1
	1 mg/kg	12.0 ± 0.5	327 ± 33	23.1 ± 4.1
	3 mg/kg	12.0 ± 0.6	347 ± 49	17.9 ± 3.5
	6 mg/kg	12.6 ± 0.4	404 ± 47	24.1 ± 4.9
	10 mg/kg	12.6 ± 0.4	390 ± 41	20.5 ± 3.8
**Nicotine**	Vehicle	12.0 ± 0.7	367 ± 82	16.6 ± 2.5
	0.05 mg/kg	14.1 ± 0.6 **	558 ± 64	16.4 ± 1.3
	0.1 mg/kg	14.4 ± 0.6 **	656 ± 76 *	19.8 ± 2.6
	0.2 mg/kg	15.7 ± 0.6 **	861 ± 114 **	33.3 ± 3.8 **
	0.4 mg/kg	16.3 ± 0.8 **	1052 ± 141 **	37.2 ± 5.4 **
**Atomoxetine**	Vehicle	11.8 ± 1.0	380 ± 115	18.4 ± 5.1
	0.1 mg/kg	10.3 ± 0.8	246 ± 58	11.1 ± 1.3
	0.5 mg/kg	8.8 ± 0.9 **	173 ± 53 *	8.8 ± 1.5 *
	1 mg/kg	8.1 ± 0.6 **	118 ± 22 **	9.5 ± 1.5 *
	2 mg/kg	7.5 ± 0.5 **	97 ± 18 **	8.6 ± 1.9 *

Data is presented as means ± SEM for break point, number of active lever presses, and session duration (min) made by rats following pretreatment with AMP (0.03–0.6 mg/kg), MPH (1–10 mg/kg), NIC (0.05–0.4 mg/kg), and ATX (0.1–2 mg/kg) in a progressive ratio task. *P < 0.05 vs. vehicle pretreatment. **P < 0.01 vs. vehicle pretreatment.

#### Methylphenidate

There was no significant effect of treatment on the number of lever presses (F4,28 = 2.2; P=0.1; η_p_^2^ = 0.24), break point (F4,28 = 1.7; NS; η_p_^2^ = 0.19), or total session duration (F4,28 = 1.4; NS; η_p_^2^ = 0.17) following methylphenidate (1–10 mg/kg) pretreatment although there was a trend to increase the number of lever presses at the 6–10 mg/kg dose range. See **Table 4**.

#### Nicotine

NIC (0.05–0.4 mg/kg) had a significant main effect of treatment on the number of lever presses (F4,44 = 13.0; P < 0.001; η_p_^2^ = 0.54), break point (F4,44 = 13.2; P < 0.001; η_p_^2^ = 0.54), and total session duration (F4,44 = 9.4; P < 0.001; η_p_^2^ = 0.46). On each measure nicotine produced a dose related increase in responding, increasing the number of lever presses, the break point and consequently session duration. Significant differences relative to control were evident at 0.1–0.4 mg/kg doses. See **Table 4**.

#### Atomoxetine

A significant main effect of atomoxetine (0.1–2 mg/kg) on the number of lever presses (F4,28 = 6.0; P=0.001; η_p_^2^ = 0.46), break point (F4,28 = 13.1; P < 0.001; η_p_^2^ = 0.65) and total session duration (F4,28 = 3.3; P < 0.05; η_p_^2^ = 0.32) was recorded. On each measure atomoxetine produced a dose related decrease in responding, decreasing the number of lever presses, reducing the break point and consequently the session duration. Significant differences relative to control were evident at 0.5–2 mg/kg doses. See **Table 4**.

### Measurement of Locomotor Activity

#### d-Amphetamine

**Figure 8:**
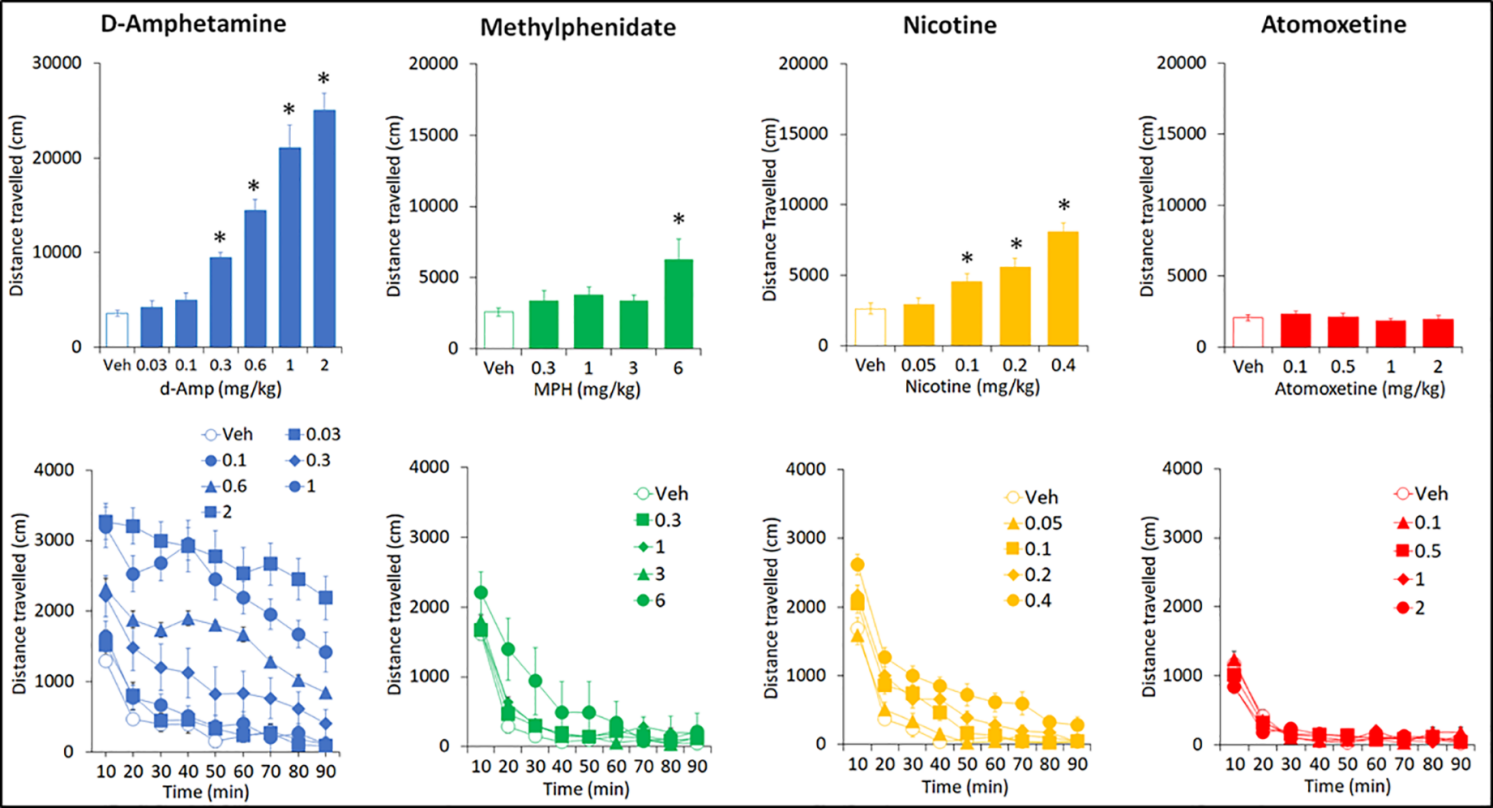
Effect of amphetamine (AMP), methylphenidate (MPH), nicotine (NIC), and atomoxetine (ATX) on locomotor activity in an open-field apparatus. Effect of AMP (0.03–2 mg/kg), MPH (0.3-6 mg/kg), NIC (0.05-0.4 mg/kg) and ATX (0.1-2 mg/kg) on locomotor activity (measured as distance traveled in an open-field apparatus) task. N=15 Long Evans rats per drug. For each drug study, all rats received each treatment in a balanced design (see Methods for details). The upper panels show the total distance traveled over the 90-min test session. The lower panels show the same data expressed by each 10 min bin to give a time course analysis. *P < 0.05 vs. vehicle pretreatment (Dunnett’s test following significant ANOVA).

AMP (0.03–2 mg/kg IP) produced a dose related increase in total distance traveled (F6,84 = 46.1; P < 0.001; η_p_^2^ = 0.77), rearing counts (F6,84 = 13.3; P < 0.001; η_p_^2^ = 0.49) and ambulatory counts (F6,84 = 40.8; P < 0.001; η_p_^2^ = 0.74) relative to vehicle pretreated controls. The threshold dose for these effects was 0.3 mg/kg. Time course analysis of the distance traveled measure showed that these differences were essentially consistent across each time bin. Occasional drug free exposures to the activity chambers were interpolated into this study to minimize complications of conditioned hyperactivity. Consequently a baseline (drug free) activity measure taken pretesting and immediately posttesting revealed no significant difference (Distance traveled: pretest: 3320 ± 305, posttest: 4547 ± 559; NS) (see **Figure 8**).

#### Methylphenidate

MPH (0.3–6 mg/kg IP) produced a dose related increase in total distance traveled (F4,56 = 3.4; P=0.01; η_p_^2^ = 0.20), rearing counts (F4,56 = 4.2; P < 0.01; η_p_^2^ = 0.23) and ambulatory counts (F4,56 = 3.9; P < 0.01; η_p_^2^ = 0.22) relative to vehicle pretreated controls. On each measure, only the 6 mg/kg significantly differed from vehicle pretreatment. Time course analysis of distance traveled measure showed the increased activity produced by MPH (6mg/kg) was limited to the initial 30 min of the test session (see **Figure 8**). At the completion of the dose-response study, a 10-mg/kg MPH dose was administered as a single dose. This treatment produced a highly significant increase in distance traveled compared to vehicle (veh: 2573 ± 291; MPH 10 mg/kg: 57576 ± 6116; P < 0.001).

#### Nicotine

NIC (0.05–0.4 mg/kg SC) produced a dose related increase in total distance traveled (F4,56 = 22.7; P=0.01; η_p_^2^ = 0.62), rearing counts (F4,56 = 13.7; P=0.01; η_p_^2^ = 0.49) and ambulatory counts (F4,56 = 24.1; P=0.01; η_p_^2^ = 0.63) relative to vehicle pretreated controls. The threshold dose for these effects was 0.1 mg/kg. Time course analysis of distance traveled measure showed the nicotine hyperactivity was apparent for at least the first 50 min of the test session (see **Figure 8**).

#### Atomoxetine

ATX (0.1–2 mg/kg IP) had no effect on total distance traveled (F4,56 = 0.8; NS; η_p_^2^ = 0.06), rearing counts (F4,56 = 0.3; NS; η_p_^2^ = 0.02) and ambulatory counts (F4,56 = 1.1; NS; η_p_^2^ = 0.07) relative to vehicle pretreated controls. Time course analysis of distance traveled measure also revealed no treatment effect by time (see **Figure 8**).

### Measurement of Plasma Levels of AMP, MPH, NIC, and ATX

Based on the pretreatment times used for these experiments, plasma samples were collected at timepoints corresponding to 0.5, 1, and 2 h into tests. To control for potential food effect on drug DMPK property, all studies were conducted in the test subjects under their normal restricted food regimen, at a timepoint distinct to behavioral testing.

#### d-Amphetamine

Increasing doses of AMP over the range 0.03–0.6 mg/kg produced related increases of drug in plasma. Exposure over the 0.1–0.3 mg/kg IP dose range (at which most effects relevant to attention and impulsivity were noted) at timepoints corresponding to behavioral testing was in the range 35–97 ng/ml (see **Table 5**).

**Table 5:** Plasma exposure levels for amphetamine, methylphenidate, nicotine, and atomoxetine at timepoints relevant to the tests.

		Plasma concentration (ng/ml)		
		0.5 h	1 h	2 h
**d-Amphetamine**	0.03 mg/kg	8 ± 2	5 ± 1	3 ± 1
	0.1 mg/kg	35 ± 5	25 ± 5	18 ± 6
	0.3 mg/kg	97 ± 20	55 ± 12	29 ± 5
	0.6 mg/kg	250 ± 23	135 ± 23	80 ± 20
	1 mg/kg	29 ± 9	20 ± 5	11 ± 1
**Methylphenidate**	3 mg/kg	113 ± 59	120 ± 55	50 ± 15
	6 mg/kg	220 ± 100	160 ± 60	70 ± 30
	10 mg/kg	600 ± 250	320 ± 150	216 ± 100
****	0.05 mg/kg	9 ± 1	7 ± 1	3 ± 1
**Nicotine**	0.1 mg/kg	15 ± 1	14 ± 2	6 ± 1
	0.2 mg/kg	37 ± 2	27 ± 1	13 ± 1
	0.4 mg/kg	60 ± 6	63 ± 18	21 ± 2
****	0.1 mg/kg	3 ± 1	2 ± 1	1 ± 1
**Atomoxetine**	0.5 mg/kg	12 ± 2	12 ± 1	6 ± 1
	1 mg/kg	23 ± 9	15 ± 4	9 ± 6
	2 mg/kg	35 ± 9	31 ± 19	24 ± 16

Both d-Amphetamine and nicotine were administered 10 min before test, and methylphenidate and atomoxetine administered 30 min before test. The timepoints of 0.5, 1, and 2 h refer to exposure levels at these stages of test session. The duration of test sessions ranged from 0.25–0.5h (progressive ratio test), to 0.5–0.45 h (usITI, sSD, Go-NoGo tests), to 1 h (long ITI 5-CSRTT). For comparison, plasma levels of d-Amphetamine, methylphenidate, nicotine, and atomoxetine reported in humans are: d-amphetamine (30–80 ng/ml; see Adderall XR Product Monograph), methylphenidate (5–20 ng/ml; see Swanson and Volkow, 2001; Storebø et al., 2015), nicotine (10–50 ng/ml; Russell et al., 1975; Matta et al., 2007), and atomoxetine (Hazell et al., 2009). Thus, there is good translation between plasma levels in rats and humans based on therapeutic effects for both d-amphetamine and nicotine. Some differences may be evident for both methylphenidate and atomoxetine, both of which also seemed to show a wider interanimal variability in terms of exposure compared to d-amphetamine and nicotine.

#### Methylphenidate

Increasing doses of MPH over the range 1–10 mg/kg produced related increases of drug in plasma although some variability between rats seemed evident. Exposure over the 3–6 mg/kg IP dose range (at which most effects relevant to attention and impulsivity were noted) at timepoints corresponding to behavioral testing was in the range 106–224 ng/ml (see **Table 5**).

#### Nicotine

Increasing doses of NIC over the range 0.05–0.4 mg/kg produced related increases of drug in plasma. Exposure over the 0.1–0.2 mg/kg SC dose range (at which most effects relevant to attention and impulsivity were noted) at timepoints corresponding to behavioral testing was in the range 15–40 ng/ml (see **Table 5**).

#### Atomoxetine

Increasing doses of ATX over the range 0.1–2 mg/kg produced related increases of drug in plasma. Exposure over the 0.5–1 mg/kg IP dose range (at which most effects relevant to attention and impulsivity were noted) at timepoints corresponding to behavioral testing was in the range 10–30 ng/ml (see **Table 5**).

## Discussion

### Rationale for Test Selection

The adoption of tests that can be used across species from the preclinical to the clinical setting is widely recognized as a logical approach to improve translational reliability (e.g. Pangalos et al., 2007; Day et al., 2008; Markou et al., 2009; Goetghebeur and Swartz, 2016; McArthur, 2017; Robbins, 2017). Attention is commonly measured using the 5-CSRTT, a test that can be conducted in rodents, primates and humans (Robbins, 2002; Bari et al., 2008; Higgins and Silenieks, 2017). A strength of the 5-CSRTT is the capability to modify task conditions to challenge attention, speed of responding and response control. For example, reducing stimulus salience by shortening its duration, or predictability by varying its rate of presentation, can be used to tax attentional demand. Conversely lengthening the time to successive stimulus presentations challenges response control, and consequently a means to evaluate impulsive action, operationally defined as premature responses made prior to stimulus onset. Extending trial number and measuring performance over trial blocks can assess vigilance, and interpolation of distractor stimuli, (e.g., brief bursts of white noise or flashing lights) have been used to challenge divided attention (Bari et al., 2008; Amitia and Markou, 2011; Higgins and Silenieks, 2017).

In the present 5-CSRTT experiments we focussed on the sITI and sSD variants to challenge attention, and a long (10-s) ITI schedule specifically to challenge response control. Tests of reproducibility confirmed a stability to deficits in discriminative accuracy and identified conditions amenable to repeated drug testing. By way of contrast, the impact of distractor stimulii seem to rely on their novelty and unpredictable property and we observe both within- and between-session habituation which renders this manipulation best suited to between subjects designs. Thus although a heightened distractability is recognized as a symptom of ADHD, we avoided the use of distractor stimuli in these experiments.

Impulsivity can be broadly subcategorized into motor (impulsive action) and decisional (impulsive choice) domains (Evenden, 1999; Dalley et al., 2011; Winstanley, 2011). Impulsive action is characterized by acting prematurely, or failing to inhibit responding, and is frequently measured as premature responding on the 5-CSRTT (Robbins, 2002; Bari et al., 2008; Higgins and Silenieks, 2017) or as inappropriate responding (i.e. false alarms) during the NoGo phase of a Go-NoGo schedule (Kolokotroni et al., 2011). Together these two tasks differentially tax aspects of motor impulsivity and have been used for this purpose in both a preclinical and clinical context (Dalley et al., 2011; Winstanley, 2011). For this reason both tasks were included in the current test platform. Finally, since motivation for the primary reward of these tasks is a critical determinant in overall performance, as is efficient control of motor function, the effect of each drug on responding for food under a progressive schedule of reinforcement (Hodos, 1961; Der-Avakian et al., 2016), and locomotor activity, was also assessed.

### Rationale for Study Design

Typically, preclinical research studies are conducted on a single occasion in experimentally/drug naïve test subjects and not subject to repetition, i.e. classical block design. On occasion, this has led to issues of experiments being underpowered and concerns about overinterpretation and reproducibility of study outcomes (Kilkenny et al., 2009; Button et al., 2013; Bespalov and Steckler, 2018). The present five-choice and Go-NoGo studies were initially run using such a block design. However, in the event of a finding considered to be of interest, e.g. a treatment effect on accuracy, the experiment was repeated in a different rat cohort and the data pooled to increase the overall sample size based on a standardized test design and dosing schedule. Such an adaptive sequential study design has been proposed as a means to improve experimental efficiency (Neumann et al., 2017). A potential disadvantage of this approach was an age difference between study subjects (typically 2–6 months but most extreme estimated to be 10–12 months) and differences in both testing and drug treatment history. However, an alternative view is that a mixed drug history and age range better reflects the human population to which these studies are being aligned. One might further argue that any positive (or null/negative) drug effect measured across a study population comprised of somewhat mixed age, pretreatment history and test experience may translate better to the clinical situation. In sum, sequential study designs as used in the present studies have been proposed as a means to increase the predictive validity of preclinical experiments (Neumann et al., 2017) and thus considered to be a valid approach in the present studies.

Two necessary procedural aspects to this study was that all experiments were conducted in adult male rats which were singly housed throughout the study. Clinically, gender differences have been noted in ADHD (see section 4.6) and so the sex bias of the present study due to logistical constraints should be recognized. A similar logistical constraint, necessitated by controlling access to food, required the rats to be singly housed throughout the study duration. Postweaning social isolation can have significant effects on behavior including that related to motivation and cognitive function relative to group housed subjects, although isolation at adulthood is less impactful (Robbins et al., 1996; Fone and Porkess, 2008; Robbins, 2016). All rats entered the present study at adulthood, and were handled daily for testing or husbandry purpose. Both of these factors are likely to negate any deleterious effects of isolation (Fone and Porkess, 2008). Also, it is important to point out that all study rats were singly housed thus controlling for any variable of housing condition.

### Characterization of Performance Across the Five-Choice Challenge Tasks

Under standard task conditions of 0.75-s SD, 5-s ITI, 100 trials, well trained rats typically responded with 90% accuracy and <10% premature responses which clearly places constraints for the assessment of drug treatments designed to improve attention or response control. Variations to stimulus duration (i.e. varying SD between 0.03 and 1 s) or predictability (i.e. varying ITI between 2 and 10 s) reduced accuracy in a reliable manner to levels as low as 10%–20% (using % hit measure in “poor” performers, sITI) and premature responses to as high as 130% (using % premature response measure in “high” impulsives, 10-s ITI), so increasing the dynamic range for pharmacological investigation.

In both the sITI and sSD schedules, omissions were related to task difficulty and for this reason we typically measured accuracy as % hit, accommodating both correct and incorrect responses, and omissions, i.e. errors of commission and omission (Robbins, 2002). The precise cause of omission errors is often interpreted as outcomes of motivational or sensorimotor deficiencies, which in many instances is probably correct (Robbins, 2002; Bari et al., 2008; Higgins and Silenieks, 2017). However, the highly significant relationship between omissions and task difficulty seen in the sITI and sSD schedules suggested to us that in this instance they reflected attentional lapses. Thus, % hit was used as the primary measure of accuracy for these two schedules.

Subjects run under the sITI schedule showed a wide continuum of accuracy under the 2-s ITI (2%–70%), while at the 5-s ITI run within the same schedule, performance was more consistent (43%–88%). Ranking animals based on % hit measure at the 2-s ITI, enabled the categorization of subjects into “low” and “high” performers based on “low” and “high” tertiles. These tertile groups were notable for three features. Firstly, there was a consistency of performance across multiple exposures to the sITI schedule, and secondly cosegregating with the % hit measure were omissions and response speed, i.e. the “low” performers had higher omissions and were slower to make a correct response. Third, the similar performance level of both tertile groups at the 5-s ITI suggested motivation was not necessarily a factor in performance.

Shortening the stimulus duration while leaving ITI constant at 5 s (i.e. sSD schedule) similarly resulted in a decline in accuracy. Ranking the animals into “low” and “high” performers based on % hit measure at the 0.03-s SD, revealed “low” performers to have higher omissions compared to their “high” counterparts. An interesting feature to emerge from this analysis however was that performance under the sSD did not predict performance under the sITI. That is, in animals run concurrently between both schedules, there was no significant correlation between performance in each task variant, using % hit as the dependent measure. Also, unlike the sITI, slower response speed did not cosegregate with lower accuracy. This suggests a distinct neuropsychological basis for performance between the sITI and sSD schedules.

Due to the relatively short ITI (≤5 s) utilized in the sITI and sSD schedules, the level of premature responses made in each schedule was low (i.e. ≤10%). However, lengthening the ITI from 5 to 10 s produced a dramatic increase in premature responses, a consistent finding that has been widely reported (Robbins, 2002; Blondeau and Dellu-Hagedorn, 2007; Higgins and Silenieks, 2017; Barlow et al., 2018). Accuracy was also reduced at the longer ITI compared to 5-s ITI, although whether this is a consequence of the lower SD (5-s ITI SD=0.75 s; 10-s ITI SD=0.3 s), or longer ITI, or both, is unclear from the present experiments.

Subgrouping rats under the 10-s ITI schedule based on the level of premature responses into “Low” and “High” impulsives (LI vs. HI) highlighted the wide range of responders on this parameter (Dalley et al., 2007; Barlow et al., 2018). Importantly there was a reasonable consistency of performance on this measure over repeated tests. Cosegregating with the LI and HI phenotype was response speed, % correct and trial number, with the HI group having faster response latencies, initiating more trials (a premature response does not constitute a trial), and a slightly lower choice accuracy. The high incidence of premature responses made by the HI relative to the LI cohort raises the likelihood that a proportion would occur coincidently with the stimulus onset. In that event there would be a high probability (80%) that they would be classified as incorrect choices, and this may account, at least in part, for the lower accuracy of the HI cohort.

Of final note, the performance of all rats under the 10-s ITI was compared to their performance under standard test conditions, and despite premature responses being significantly lower at the 5-s ITI, the HI rats had significantly higher premature responses compared to LI rats. This supports the view of an impulsive phenotype that can be detected under multiple test conditions even under standard conditions used for training.

### Characterization of AMP, MPH, ATX and NIC on Performance

An important objective of this work was to characterize the profiles of AMP (Adderall^®^), MPH (Ritalin^®^), ATX (Strattera^®^), and NIC in these tasks. Although each drug has been widely reported on 5-CSRTT performance by various groups (see subsequent references), an advantage of the present work is that each have been tested under equivalent test conditions (see **Table 6** for Summary). This eliminates variations in environmental and precise test conditions used between labs, and that is often cited as a factor influencing robustness of behavioral research findings across labs (Bespalov et al., 2016; Voelkl and Würbel, 2016; Bespalov and Steckler, 2018). Measured over increasing dosage, the psychostimulants AMP and MPH elicit a continuum of behavioral and cognitive activation which transition from the beneficial to the detrimental (Grilly and Loveland, 2001; Wood et al., 2013; Berridge et al., 2012). Therefore, care was taken to avoid the overt motor stimulant doses of both drugs which are associated with their abuse potential and disruptive effects on behavior. While mild increases in distance traveled and rearing counts were noted at the highest doses of AMP (0.3 mg/kg), MPH (6 mg/kg) and NIC (0.1–0.2 mg/kg) tested in the 5-CSRTT, these were of a magnitude reflective of a moderate arousal, and considered beneficial to cognitive performance (Grilly and Loveland, 2001; Wood et al., 2014; Berridge et al., 2014). AMP doses in excess of 0.6 mg/kg produced a state of hyperarousal which would likely prove detrimental to task performance (Grilly and Loveland, 2001; Wood et al., 2014).

**Table 6:** Summary of profiles for amphetamine (AMP), methylphenidate (MPH), nicotine (NIC), and atomoxetine (ATX) in tasks designed to measure attention and impulsivity and motivation for food reinforcement.

Drug	Dose (mg/kg)	Five-choice serial reaction time task			Go/NoGo task		Progressive Ratio
		sITI	sSD	Long 10-s ITI	Go	NoGo
**d-Amphetamine**	**0.03–0.6**	(↑Atn)/(↑I)^1^	(↔Atn)/(↑I)	(↔ Atn)/(↑I)	(↔Acc)	(↑Acc)/(↓I)	(↑BP)
**Methylphenidate**	**1–6**	(↑Atn)/(↑I)^1^	(↔Atn)/(↔I)	(↔ Atn)/(↓↑I)^2^	(↔Acc)	(↑/↔Acc)/(↓/↔I)^3^	(↔BP)
**Nicotine**	**0.05–0.4**	(↑Atn)/(↑I)	(↑Atn)/(↑I)	(↑Atn)/(↑I)	(↔Acc)	(↔Acc)/(↔I)	(↑BP)
**Atomoxetine**	**0.1–1**	(↓Atn)/(↓I)^4^	(↔Atn)/(↓I)^4^	(↔Atn)/(↓I)^5^	(↔Acc)	(↑Acc)/(↓I)	(↓BP)

(Atn) = attention (five-choice task: % correct or % hit measure); (I) = impulsivity (five-choice task, Go-NoGo task, delay discounting task as appropriate); (Acc) = choice accuracy (Go-NoGo task).

(BP) = Break point for food reinforcement in the Progressive ratio task.

(↑) = improvement/increase; (↓) = impairment/decrease; (↔) = no effect.

1 Effect of AMP and MPH to improve attention (Hit rate and response speed) was in low performing subgroup.

2 Effect of MPH on premature responses differed according to baseline, i.e. reduced prematures in HI, and increased prematures in LI subgroups.

3 Trend to decrease false alarms.

4 ATX reduced accuracy in the short intertrial interval (sITI) and reduced overall trials in the short stimulus duration (sSD) schedule. These effects were most notable in “High” performing groups.

5 Effect of ATX on premature responses was most evident in HI subgroup.

In terms of attentional accuracy, NIC produced the most robust effects, significantly improving choice accuracy measured either as % correct or % hit across all three 5-CSRTT challenge formats (e.g. see also Mirza and Stolerman, 1999; Grottick and Higgins, 2000; Grottick and Higgins, 2002; Hahn et al., 2002; Day et al., 2007; Young et al., 2013). The profiles of the psychostimulant drugs AMP and MPH on accuracy measures recorded in the 5-CSRTT were somewhat similar and more restricted compared to NIC. Both drugs improved attentional performance in the sITI, yet neither improved accuracy under the sSD and long 10-s ITI condition. Tested under the sITI condition, AMP (0.1–0.3 mg/kg) and MPH (3–6 mg/kg) improved attentional accuracy (measured either as % correct or % hit rate), increased speed of responding and reduced missed trials (see Grottick and Higgins, 2002; Bizarro et al., 2004; Andrzejewski et al., 2014; Slezak et al., 2018). This proattentive effect was particularly evident in the poorly performing cohort (see also Puumala et al., 1996; Robinson, 2012; Tomlinson et al., 2014; Turner and Burne, 2016; Caballero-Puntiverio et al., 2017; Caballero-Puntiverio et al., 2019). Differences between the two stimulant drugs became evident on measures of motor impulsivity, for while AMP increased premature responses in the 5-CSRTT (all conditions) and reduced false alarms in the Go-NoGo task, MPH only trended to a similar effect. Indeed in the long ITI condition the effect of MPH was baseline dependent, increasing premature responses in LI, and decreasing this measure in HI (see also Puumala et al., 1996; Fernando et al., 2012; Tomlinson et al., 2014; Caprioli et al., 2015). The bidirectional profile for impulse related measures affected by AMP between the 5-CSRTT and Go-NoGo tests highlights the multi-faceted nature of impulsive action and the necessity for specific tasks to differentiate between them (Dalley et al., 2011; Winstanley, 2011).

As an approved treatment for ADHD along with formulations of AMP and MPH, the profile of ATX across the 5-CSRTT schedules was very distinct. In contrast to both AMP and MPH, ATX did not improve accuracy in the sITI schedule, rather it detrimentally affected performance particularly in the high performing subgroup. Indeed, ATX failed to produce a significant improvement in accuracy (measured either as % correct or % hit) under any 5-CSRTT schedule or in any attentional subgroup. Positive effects of ATX on attentional measures have been reported in some 5-CSRTT studies typically under conditions of delayed ITI (see Navarra et al., 2008; Baarendse and Vanderschuren, 2012; Paterson et al., 2012; Callahan et al., 2019), and in low attentive subgroups (Robinson, 2012; Tomlinson et al., 2014). However, these findings are inconsistent (e.g. Blondeau and Dellu-Hagedorn, 2007; Robinson et al., 2008; Fernando et al., 2012; Ding et al., 2018) and in the present series of experiments we could not find evidence for a proattentive effect of ATX pretreatment, even in low performing subgroups. At the tested dose range, ATX (0.1–2 mg/kg) had no significant effect on locomotor activity.

The most reliable effects of ATX were recorded on measures of impulsive action with ATX reliably decreasing premature responses in each 5-CSRTT schedule, notably the long ITI variant, and also false alarms in the Go-NoGo task with a consequent improvement in overall accuracy. Positive effects of ATX on premature responses across various 5-CSRT task variants have been widely reported, occasionally with a concomitant slowing of response speed and increased omissions (e.g. Blondeau and Dellu-Hagedorn, 2007; Navarra et al., 2008; Robinson et al., 2008; Baarendse and Vanderschuren, 2012; Fernando et al., 2012; Paterson et al., 2012; Robinson, 2012; Tomlinson et al., 2014; Callahan et al., 2019). Similar to the present findings, the effects of ATX on premature responding may be most apparent in test subjects identified as high impulsive (Fernando et al., 2012; Tomlinson et al., 2014). While improved response control has been reported for ATX on a stop-signal task of impulsive action (Robinson et al., 2008), and in a rodent CPT task (Tomlinson et al., 2014; Ding et al., 2018; Caballero-Puntiverio et al., 2019) we are unaware of equivalent findings reported on false alarms measured in a rodent Go-NoGo task. Thus, the present studies extend the positive characterization of ATX to a further measure of impulsive action.

Measures of plasma exposure taken from study subjects at timepoints corresponding to test, revealed equivalence to human exposures for some, but not all the test drugs. The majority of AMP effects were evident at 0.1–0.3 mg/kg, corresponding to a low dose range (Grilly and Loveland, 2001) and plasma [drug] levels of 35–95 ng/ml, which is in reasonable agreement to therapeutic levels of AMP attained by various formulations (e.g. 30–80 ng/ml; see Adderall XR Product Monograph, 2017) (see also Slezak et al., 2018). Similarly NIC exposure in the plasma compartment at the efficacious dose range (0.1–0.2 mg/kg) was equivalent to human plasma levels recorded in moderate smokers (15–40 ng/ml; see Russell et al., 1975; Matta et al., 2007). However, for ATX, plasma exposure over the 0.5–1 mg/kg IP dose range (at which most effects relevant to attention and impulsivity were noted) was in the range 10–30 ng/ml which is lower than the human therapeutic plasma concentration of ATX (range 300–600 ng/ml) (Hazell et al., 2009). Species differences in plasma protein binding and thus free plasma concentration may in part account for this difference.

Some differences between preclinical and clinical exposures also seemed evident with MPH. Increasing doses of MPH over the range 1–6 mg/kg produced dose-related increases of plasma [drug] over the range 30–220 ng/ml (see also Shimizu et al., 2019 for comparison). The human therapeutic plasma concentration of MPH is in the range 10–40 ng/ml (Swanson and Volkow, 2001; Storebø et al., 2015), somewhat lower than that identified in the present studies. Berridge and colleagues (Berridge et al., 2012; Spencer et al., 2014) highlight a spectrum of cognitive effects of psychostimulants including MPH with distinct dose profiles. Thus, a low MPH dose of 0.5 mg/kg (IP route) with concomitant plasma exposure within the clinical range, may be optimal for working memory improvement, yet suboptimal for proattentional effect, where they report a maximal improvement at 2 mg/kg—overlapping with the present findings. These workers propose that the lower (0.5 mg/kg) dose of MPH corresponding to a clinically relevant exposure, represents an optimal procognitive dose, devoid of the stimulant effects evident at higher doses corresponding to supratherapeutic exposures. In our experience, MPH doses of 10 mg/kg and above (MPH [plasma] >300 ng/ml) are necessary to elicit overt motor stimulation and disruption of complex behavioral processes required for 5-CSRTT performance. Therefore, the present data suggest that in rodent, positive effects of MPH in tasks relevant to attention and impulsive action do extend to the 1–6 mg/kg range (see also Bizarro et al., 2004; Navarra et al., 2008; Tomlinson et al., 2014).

### Pharmacological Considerations

AMP, MPH, ATX, and NIC each had a distinct profile across the various tests, which is a likely reflection of their distinct pharmacological property. Through inhibition of the catecholamine reuptake transporters, both AMP and MPH increase synaptic levels of DA and NA in cortical subregions (Heal et al., 2009; Spencer et al., 2014), a feature believed critical to the therapeutic efficacy of ADHD drugs (Arnsten, 2009; Berridge et al., 2012; Bolea-Alamañac et al., 2014; Spencer et al., 2014). AMP has the additional property of directly enhancing release of DA from vesicular stores (Daberkow et al., 2013; Heal et al., 2013; Hutson et al., 2014) which likely accounts for its greater effect on DAergic function relative to MPH, especially in subcortical regions such as the striatum/accumbens (Kuczenski and Segal, 1997; Kuczenski and Segal, 2001; Heal et al., 2009). In such instances where the effects of both drugs on behavior differ, for example, the measure of premature responding recorded in the five-choice task, may reflect differences in the magnitude of change, or balance between, NA and DA neurotransmission. Thus elevated DA tone within structures innervated by the mesolimbic system will result in behavioral disinhibition, including increased premature responses (Robbins, 2002; van Gaalen et al., 2006).

Elevations of central NA function through either selective reuptake inhibition (atomoxetine, reboxetine) or alpha 2A agonists such as guanfacine have been reported to reduce measures of impulsive action recorded in the five-choice and stop-signal tasks (Eagle et al., 2008; Robinson et al., 2008; Pattij et al., 2011; Fernando et al., 2012; Robinson, 2012). The present findings with ATX essentially confirm this effect in the long ITI 5-CSRTT schedule, but now also extend to a go-nogo task. Similar to MPH and AMP, ATX increases extracellular levels of NA in prefrontal cortex, although in contrast it has null effect or a tendency to decrease accumbens DA release (Heal et al., 2009; Yohn et al., 2016), which may explain the property of ATX to slow response speed and dampen response output, notably under conditions of high response rate. For example, the most marked effects of ATX were apparent in the “high performer” or “high impulsive” groups in the sITI and 10-s ITI schedules respectively. Given that ATX reduced response rate and break point in a PR schedule of food reinforcement would suggest that an effect on primary motivation may account, at least in part, for these effects (Achterberg et al., 2016; Yohn et al., 2016). PET imaging studies in primate suggest that clinically relevant exposures of ATX will occupy the 5-HT as well as the NA transporter (Gallezot et al., 2011; Ding et al., 2014). Since SSRI drugs may blunt motivation (Mathes et al., 2012; Rosenberg et al., 2013; Yohn et al., 2016), an inhibitory effect of ATX at the 5-HT and/or NA transporter might contribute to this property.

The distinct profiles of AMP and ATX highlight contrasting effects of elevated DA and NA tone on impulsive behavior. MPH inhibits the reuptake of both DA and NA with approximately similar potency (Heal et al., 2009). This would imply that any effect of systemically administered MPH is dependent on the level of ongoing tone between these neurotransmitter systems, which vary according to behavioral state, level of performance or task requirement. Indeed the proattentive effects of MPH may be most evident in low performers (Puumala and Sirvio, 1998; Tomlinson et al., 2014; present study). Furthermore, under the long ITI schedule, the effect of MPH on premature responses differed between the LI and HI cohorts, reducing this measure in the HI, while increasing in the LI (see also Fernando et al., 2012; Tomlinson et al., 2014). Neurochemical differences have been reported between subgroups categorized according to phenotypic differences in attention and impulsivity (Puumala and Sirvio, 1998; Dalley et al., 2007).

Through broad activation of nicotinic cholinoceptors, NIC impacts on multiple neurotransmitter systems, including an enhancement of cholinergic and dopaminergic tone in cortical and subcortical zones (Livingstone and Wonnacott, 2009). The most parsimonious explanation for the positive effect of NIC on attentional accuracy under all the variable ITI and sSD conditions, is a direct enhancement of cholinergic tone, likely within cortical targets innervated by the ascending nucleus basalis of Meynert (NbM) pathway (McGaughy et al., 2002; Lehmann et al., 2003; Hahn et al., 2003b; Lambe et al., 2005). Conversely effects on response speed and premature responding likely reflect a well characterized effect of nicotine on subcortical DA systems *via* α_4_β_2_ nAchR activation (Grottick et al., 2003; Hahn et al., 2003a; Mohler et al., 2010; Young et al., 2013). The dissociation between nicotine and MPH/AMP across the sITI (all 3 active) and sSD (only nicotine active), was of note and supported the findings from the subgroup and correlational analysis that these task variants produce distinct attentional challenges to the test subjects with differing neuropsychological substrates.

### Translational Considerations

According to the DSM-V-TM (Diagnostic and Statistical Manual of Mental Disorders, 2013, American Psychiatric Association) ADHD may present as one of three symptom patterns: predominantly inattentive (ADHD-I), predominantly hyperactive/impulsive (ADHD-HI), and a combination of both (ADHD-C). Thus, the subgrouping of inattentive (i.e. sITI and sSD schedules) and impulsive (long 10-s ITI schedule) subgroups provide logical models of the ADHD-I and ADHD-HI conditions respectively (Tomlinson et al., 2014). Under the present test conditions, we could not find a significant population of rats that shared an inattentive-impulsive phenotype which would serve as a viable model for ADHD-C (Blondeau and Dellu-Hagedorn, 2007). Nonetheless the identification of inattentive and impulsive subgroups provides a method of generating models without any underlying assumption as to mechanism of action, which may be considered advantageous given uncertainty around the precise etiology of many neuropsychiatric conditions (Hayward et al., 2016). Furthermore, in addition to serving as models of ADHD-I and ADHD-HI, these phenotypes also enable investigation of other conditions characterized by inattention or high impulsivity such as drug abuse, OCD, schizophrenia (see Dalley et al., 2007; Hayward et al., 2016).

Based on outcomes from the present experiments, and the preclinical five-choice/CPT literature in general, one might predict that given the reliable effects of ATX on measures of motor impulsivity rather than attention, that ATX may be of most benefit in ADHD subjects categorized as ADHD-HI rather than ADHD-I. However, at the present time meta-analyses of ADHD trials do not seem to identify a particular subgroup as specifically responsive to ATX treatment (Faraone and Glatt, 2010; Asherson et al., 2014; Bolea-Alamañac et al., 2014). A similar generalization can also probably be made for AMP and MPH across ADHD subjects (Faraone and Glatt, 2010; Castells et al., 2011a; Castells et al., 2011b; Bolea-Alamañac et al., 2014; Epstein et al., 2014; Storebø et al., 2015), although in both instances positive treatment effects on attentional and impulsive measures have been reported perhaps making predictions for responsive ADHD subgroups less obvious. Improvements in both domains however may explain the higher responder rate and/or efficacy for both stimulant drugs compared to ATX as treatments for adult and juvenile forms of ADHD (Faraone and Glatt, 2010; Bolea-Alamañac et al., 2014).

At the present time, a more useful reverse translational exercise is to compare the profiles of AMP, MPH, ATX, and NIC across specific tests conducted between the preclinical and clinical context. AMP has a positive effect on CPT performance both in healthy adults and in individuals diagnosed with ADHD with positive effects on processing speed and attentional domains such as vigilance (Castells et al., 2011a; MacQueen et al., 2018). This shows a translational consistency to the rodent 5-CSRT under conditions such as variable ITI (present study; Bizarro et al., 2004) and extended trials (Grottick and Higgins, 2002). AMP has also been reported to reduce false alarms in a human Go/NoGo task (De Wit et al., 2002) which is mirrored by the current Go-NoGo experiments. Chamberlain et al. (2007) have also reported improvements in impulsive action following atomoxetine treatment in juvenile and adult ADHD individuals using the stop-signal task, thus advancing the findings for ATX conducted on rodent impulsivity tasks into the clinic.

Meta-analyses of CPT studies by Losier et al. (1996) report that MPH treatment in both adults and children with ADHD is associated with fewer commission and omission errors, and faster processing speed (see also Riccio et al., 2001a; Riccio et al., 2001b). These findings compare favorably to the observation that MPH improved performance in the sITI schedule, largely through reducing commission and omission errors and increasing response speed in low performers (see also Bizarro et al., 2004; Navarra et al., 2008; Tomlinson et al., 2014), although it must be noted the preclinical literature is inconsistent probably reflecting the importance of task variables and baseline subject performance (Fernando et al., 2012; Paterson et al., 2012; Caprioli et al., 2015; Ding et al., 2018). In a CPT study conducted in youth categorized with ADHD, Bedard et al. (2015) reported superior effect of an MPH formulation relative to ATX on sustained attention, which would certainly reflect our own observations with these drugs on rodent performance in the sITI task.

Consistent with other preclinical studies, we found NIC to reliably improve attention under various 5-CSRTT schedules (see also Mirza and Stolerman, 1998; Grottick and Higgins, 2000; Grottick and Higgins, 2002; Hahn et al., 2002; Bizarro et al., 2004; Day et al., 2007). There is also good consistency for proattentive effects of NIC in human CPT experiments conducted in smokers, individuals with psychiatric conditions and their controls. Across all groups, NIC speeds reaction time, reduces omission and commission errors and improves accuracy (e.g. Levin et al., 1996; Levin et al., 1998; Barr et al., 2008; Heishman et al., 2010; Myers et al., 2013).

While ADHD is recognized in both males and females, there may be gender differences in the clinical expression of symptoms. For example, males are more likely to exhibit symptoms of hyperactivity and lack of impulse control, and females may present with lower ratings of attention relative to males. Also ADHD is more frequently identified in males, although this may be in part linked to symptoms being more evident, especially in boys (see Gaub and Carlson, 1997; Gershon, 2002; Rucklidge, 2010). Similar to the present studies, the majority of published preclinical reports describe drug effects of AMP, MPH, ATX, and NIC on measures of attention and motor impulsivity in male rats. The study of Tomlinson et al. (2014) however does describe effects of MPH and ATX in female rats, and similar to reports in males, reliable effects of ATX on measures of impulsivity were reported. Both MPH and ATX improved attention in low attentive female rats (Tomlinson et al., 2014; see also Caballero-Puntiverio et al., 2020), effects which compare to some reports in males (Puumala et al., 1996; Navarra et al., 2008; Robinson, 2012; see also Caballero-Puntiverio et al., 2017; Caballero-Puntiverio et al., 2019). Future experiments should directly compare any proattentive effect of these drugs between male and female study cohorts, particularly in light of gender differences in ADHD symptoms. At the present time it seems there is no clear consensus regarding gender × treatment effects in humans (Rucklidge, 2010).

Taken together, these findings support the premise that endophenotypes such as attention and impulsivity can be objectively measured across the preclinical-clinical divide using appropriate tests and experimental conditions. Furthermore, there seems reasonable cross-species consistency for effect of AMP, MPH, NIC, and ATX across these domains. Assuming the animal studies are conducted with acknowledgement to appropriate study design and power, this should create confidence for the forward translation of NCE’s from the preclinical to clinical setting.

## Data Availability Statement

The raw data supporting the conclusions of this article will be made available by the authors, without undue reservation, to any qualified researcher.

## Ethics Statement

All in-life studies were conducted at the InterVivo Solutions test facility and approved by an Institutional animal care and use committee, according to guidelines established by the Canadian Council for Animal Care (CCAC).

## Author Contributions

GH wrote the manuscript. GH, AP, CMo, HL, and JB contributed conception and design of the study. LS and CMa conducted the experiments. GH and ST conducted the statistical analysis of experimental data. AP, CMo, HL, and JB contributed to manuscript revision, read and approved the submitted version.

## Funding

The authors declare that this study received funding from InterVivo Solutions Inc and H. Lundbeck A/S. The study design, analysis and interpretation of data, the writing of this article and the decision to submit it for publication represent the views of the authors and not necessarily that of the funding sources.

## Conflict of Interest

The authors declare that the research was conducted in the absence of any commercial or financial relationships that could be construed as a potential conflict of interest.

GH, LS, CMa and ST were employed by InterVivo Solutions Inc. AP, CMo, HL and JB were employed by H. Lundbeck A/S.
